# Empagliflozin in paediatric heart failure: model-based optimisation of a pharmacokinetic bridging study

**DOI:** 10.3389/fmed.2025.1522131

**Published:** 2026-02-23

**Authors:** Sebastiano A. G. Lava, Alessandro Di Deo, Salvatore D’Agate, Oscar Della Pasqua

**Affiliations:** 1 Pediatric Cardiology Unit, Department of Pediatrics, Centre Hospitalier Universitaire Vaudois and University of Lausanne, Lausanne, Switzerland; 2 Clinical Pharmacology & Therapeutics Group, University College London, London, United Kingdom; 3 Heart Failure and Transplantation, Department of Paediatric Cardiology, Great Ormond Street Hospital, London, United Kingdom; 4 Division of Clinical Pharmacology and Toxicology, Institute of Pharmacological Sciences of Southern Switzerland, Ente Ospedaliero Cantonale, Lugano, Switzerland

**Keywords:** empagliflozin, heart failure, protocol optimisation, modelling and simulation, paediatric clinical trials, repurposing, SGLT-2 inhibitors

## Abstract

**Aims:**

Current therapy for paediatric heart failure is still unsatisfactory, and trials in this population have often failed. Here we apply a model-based approach to optimise the study design and increase the probability of success of a prospective trial aimed at establishing the dose rationale for empagliflozin in children with heart failure. The proposed prospective protocol is based on the assumption that the cardioprotective mechanisms and efficacy of SGLT-2 inhibitors in the paediatric population can be extrapolated from adults.

**Methods:**

A nonlinear mixed effects modelling approach incorporating prior information from pharmacokinetics (PK) in adults was used to extrapolate empagliflozin disposition parameters to children with ≥15 kg body weight. Protocol elements of interest were sampling schedule, dose, and sample size. These features were explored using an optimization algorithm ($DESIGN) and a simulation re-estimation procedure (SSE) in a large virtual paediatric cohort, overcoming some of the difficulties associated with low parameter precision in small populations.

**Results:**

A two-compartment pharmacokinetic model with sequential zero- and first-order absorption, absorption lag time and first-order elimination was identified. Clearance and distribution parameters were assumed to vary allometrically with body weight. We defined the lowest weight of 15 kg as inclusion criterion for the prospective trial, achieving, with the lowest commercially available tablet of 10 mg, a median AUC ratio of 1.03 (interquartile range 0.82–1.30) relative to the systemic exposure observed in a 50 kg adult receiving the 25 mg dose (median 7,163, IQR 6115–8,338 nmol*h/L). An optimised sampling scheme for a study with 12 patients was selected, which includes a sampling matrix with 4 different groups. Such a design enables the characterisation of empagliflozin exposure, along with exploratory safety and efficacy data collection in the population of interest.

**Conclusion:**

Repurposing of drugs for paediatric rare diseases is fraught with challenges. Our results indicate that a weight-banded regimen with commercially available tablets of 10 mg empagliflozin can be used in a prospective protocol including paediatric patients with body weight ≥15 kg. In addition, this study illustrates the importance of optimising evidence generation in paediatric clinical trials through model-based approaches, ensuring that available knowledge is used to maximise the information content and reduce patient burden.

## 1 Introduction

The identification of genetic and non-genetic factors has contributed to further understanding of childhood diseases. Of note is paediatric heart failure, a rare condition affecting approximately 1–8 out of 100,000 children. Yet, it represents a relevant health burden, with almost 15,000 yearly hospitalisations just in the USA (1). Current therapy delivers unsatisfactory results, in-hospital (7–26%) and 5-year mortality (30–50%) still being high (2–4). Indeed, some of these children end up with mechanical assist devices and heart transplant – with the pertaining dilemma of organ shortage, implying a waiting-list mortality of 15–30% (5). Furthermore, heart transplant is not a perfect therapy, offering a median life expectancy of just ~20 years (6), pretty limited for a young child. As illustrated by recent examples in different therapeutic indications (7, 8), there is an opportunity to repurpose medications like sacubitril/valsartan and sodium-glucose cotransporter-2 (SGLT2) inhibitors from adults to children, extrapolating efficacy across populations (9, 10). The availability of suitable regimens for the paediatric population represents a shift in the paradigm for the treatment of paediatric heart failure.

In fact, there have been impressive improvements in the treatment of heart failure in adults; the most recent and striking probably being the use of dapagliflozin and empagliflozin (11–13). Indeed, since 2021, guidelines for heart failure therapy in adults have recommended either dapagliflozin or empagliflozin being added to ACE-inhibitors, beta-blockers and mineralocorticoid receptor antagonists as part of the standard clinical management, without any clear preference or advantage of one molecule over the other (14, 15). The mechanisms of cardiovascular protection of SGLT2 inhibitors are multiple and include among others: diuresis (with a differential role on intra- and extracellular body fluid compartments), with reduction of cardiac overload, suppression of sympathetic nervous system overdrive, metabolism shift towards ketone bodies positively impacting cardiac energetics, increased haemoglobin/haematocrit, modification of Na^+^/K^+^/Ca^2+^ ionic homeostasis, improvement of uric acid metabolism, and modulation of the FGF-23 / klotho pathway (16–19). Empagliflozin is well tolerated and has a favourable pharmacokinetic profile, with a half-life of 7–12 h and a t_max_ of approximately 1–2 h (18, 20).

In the past, attempts to repurpose drugs or extend the therapeutic indication for paediatric heart failure and cardiovascular diseases have often failed, mainly because of the lack of a dose rationale, insufficient sample size, inappropriate formulations, or inadequate endpoints (19, 21–23). To address these issues, we have implemented a repurposing plan, exploiting the opportunities offered by modelling, simulation and extrapolation principles (24–26). Indeed, we attempt to establish a solid pharmacological basis for the use of empagliflozin in paediatric heart failure based on the assumption that efficacy and safety can be extrapolated from adults. Specifically, we explore the use of modelling and simulation as a tool for protocol design optimisation, informing the dose, dosing regimen, sampling frequency/intervals and data analysis in prospective studies evaluating efficacy and safety in this population. The advantages of such an integrated approach for the evaluation of the pharmacokinetics, efficacy and safety of medicinal products for paediatric diseases have been highlighted for a wide range of diseases and conditions (27–31).

We aim to optimise the starting dose, sampling schedule, sample size, and number of visits for the evaluation of the pharmacokinetics and preliminary data on the efficacy of empagliflozin in prospective clinical trials in children and adolescents with heart failure. Given the lack of a dispersible tablet or liquid dosage form, only patients ≥6 years old will be considered for the proposed phase 2a protocol, i.e., patients who are able to swallow the commercially available tablets. The results of this trial will define the most appropriate dosing regimen for this population and explore the best suited endpoints to be used in subsequent efficacy studies. Hence, the current modelling, simulation and extrapolation study will inform the overall protocol design, i.e., inclusion criteria, starting dose, optimal sampling times, sample size, and number of visits, thereby minimising the number of patients and samples while maximising the reliability of the collected data (and consequently, the precision and accuracy of the estimates). We anticipate that the implementation of such an optimised protocol will reduce patient burden and improve trial feasibility.

## 2 Methods

**Figure 1:**
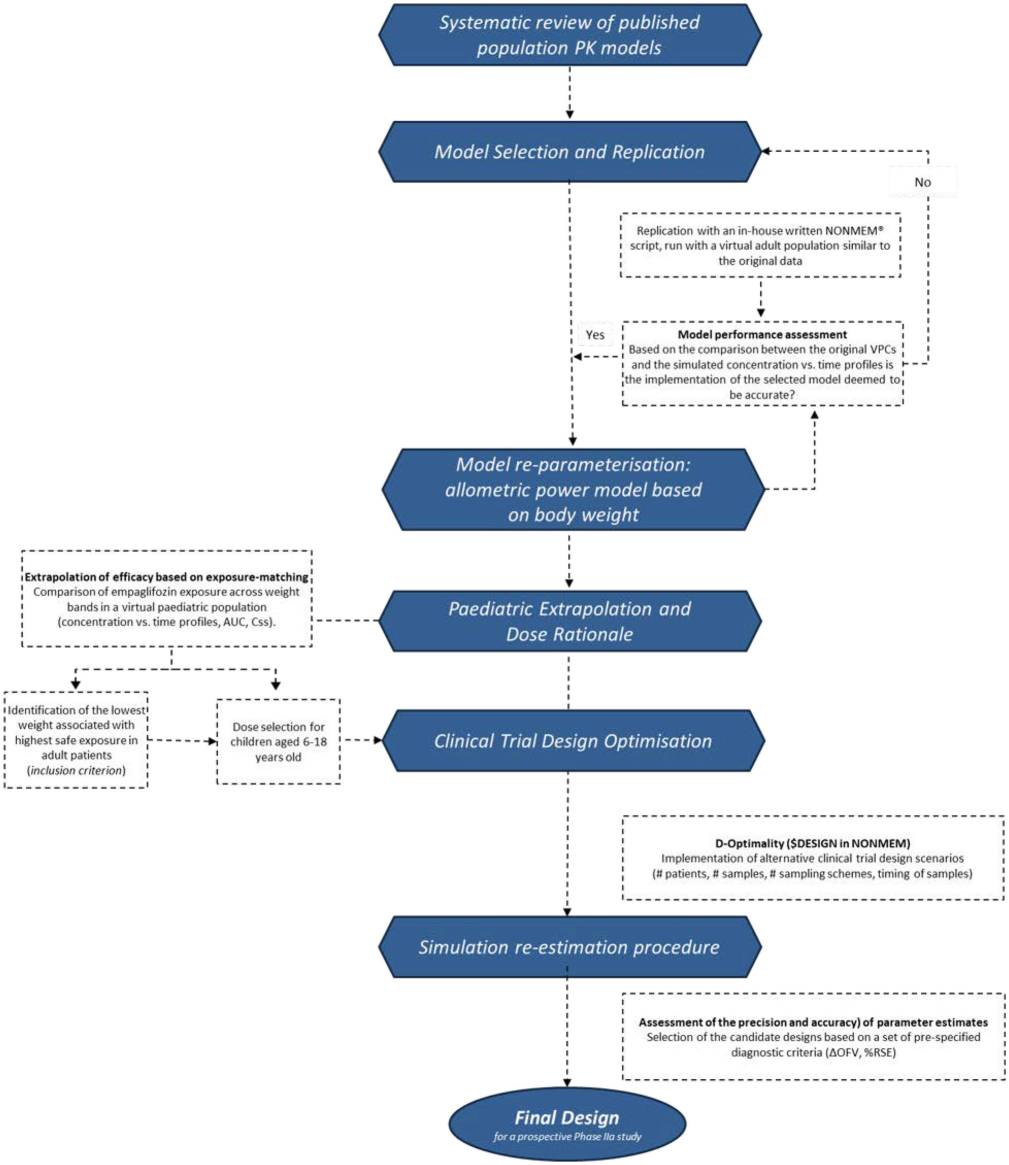
Flow-diagram describing the implementation of a model-based approach for repurposing of empaglifozin in paediatric heart failure. Given the assumption of comparable PKPD relationship in adults and children, a dose rationale is proposed which results in similar exposure across populations. To this end, the pharmaccokinetics of empaglifozin is extrapolated, taking into account the effect of body weight. Subsequently, study design is optimised to ensure reliable data supporting the proposed dosing regimen.

Given the underlying pathophysiology of heart failure, the mechanism of action of empagliflozin, and assuming that the efficacy in adults can be extrapolated to children, we show the steps required to implement a study protocol that ensures the identification and selection of a well-performing population pharmacokinetic model of empagliflozin in adults, its reparameterization applying allometric concepts and the extrapolation to a virtual paediatric population with demographic and clinical characteristics similar to the one to be included in prospective phase 2a clinical trials (Figure 1). To ensure robustness, two approaches have been considered for the purpose of design optimisation, namely D-optimality concepts based on the expectation of the determinant, and simulation-based optimisation (simulation re-estimation procedures). Importantly, we aimed to identify the best feasible, informative design from a pragmatic, clinical perspective, taking into account practical and ethical constraints.

As this article is based on *in silico* modelling and simulation, it does not include data from any new studies with human participants or animals performed by any of the authors. All clinical data used for the development of the model, as well as those required for re-sampling of the baseline characteristics of the virtual patient cohorts, which were generated for the evaluation of the different simulation scenarios described in this study, were derived from clinical trials that have been performed according to the Declaration of Helsinki and were approved by the required ethics committee(s) and/or ethics review board(s). Re-use of the anonymised data for the purpose of the current investigation is in alignment with the terms of informed consent.

### 2.1 Selection of a reference population pharmacokinetic model for empagliflozin in adults

Searches were performed in the National Library of Medicine up to February 16th, 2023. Although we decided to primarily use only one database, we followed the 2020 Preferred Reporting Items for Systematic Reviews and Meta-Analyses recommendations (32). The search string used was “empagliflozin AND pharmacokinetics.” Original reports with no date limits were contemplated. References listed within bibliographies of the included records and relevant articles known to the authors were also considered. Identified titles and abstracts were independently screened by two reviewers (SL and ADD) in an unblinded fashion. Full-text publications of candidate reports were reviewed in detail for eligibility. Any discrepancies were resolved by discussion among authors until consensus was reached.

We included original articles reporting population pharmacokinetic models of empagliflozin in humans. Reports in languages other than English, French, German, Italian, Portuguese, or Spanish were excluded. Similarly, articles reporting non-compartmental pharmacokinetic analyses, or not reporting a pharmacokinetic model (i.e., papers focusing exclusively on pharmacodynamics) were excluded. Data were collected according to a pre-defined checklist, including demographics, studied disease, sample size, investigated dose(s), modelling software employed, used validation methods, pharmacokinetic parameters, included covariates explaining interindividual variability, and residual error.

To qualify as a reference model for the subsequent modelling steps, the following criteria had to be fulfilled: (1) sufficient details provided in order to replicate the model, (2) model validation including visual predictive checks (VPCs), (3) accurate replication of modelling results on a virtual population with similar characteristics as the one on which the model was originally developed. Demographic and other relevant baseline characteristics (i.e., age, sex, weight, body mass index (BMI), and creatinine levels and/or estimated glomerular filtration rate (eGFR)) required to build the virtual population used in the evaluation of the predictive performance of the model were retrieved from the CDC-National Health and Nutrition Examination survey (NHANES) database (33).

### 2.2 Scaling of pharmacokinetics and prediction of exposure in virtual paediatric patients

Following model screening (34–39), the selected model was reparameterised taking into account the role of developmental growth on drug disposition (24).

To ensure the inclusion of relevant covariates during the assessment of the dose rationale for empagliflozin in children, anonymised clinical characteristics from a small representative sample of the heart failure paediatric population followed by some of us (Table 1) were used as basis for resampling and creation of a virtual population similar to the one to be included in the prospective phase 2a trial. Ultimately, as renal function maturation is complete within 1 to 2 years after birth, only body weight was identified as a significant covariate in this model. Consequently, a large virtual cohort was simulated based on normally distributed weights, with mean and distribution (38.1 ± 16.8 kg) reflecting the actual clinical population characteristics. Random sampling was then used to optimise sampling procedures, assuming study protocols with 12, 18 and 40 patients. Doses and dosing regimens were assessed based on weight bands, as a dose in mg/kg was deemed not feasible for the available dosage form (i.e., tablets).

**Table 1:** Baseline characteristics of the population supporting model parameter estimation and subsequent simulation scenarios described in the current study.

	Mondick et al. (36)	Survey among (*n* = 5) paediatric heart failure patients followed at our hospital	Adult virtual population (NHANES)	Paediatric virtual population (NHANES)
Age [years]	41 [34–48]	13 [12.7–13.1]	41 [29–53]	12 [8–15]
Weight [kg]	79 [70–89]	42 [27–44]	75 [65–85]	48 [32–64]
Height [cm]	176 [163–190]	163 [149–164]	167 [160–175]	152 [134–164]
BMI [kg/m^2^]	25.6 [23.1–28.1]	16.3 [15.5–17.8]	26.7 [23.6–30.0]	20.1 [17.0–24.2]
Sex (M:F)	M 53 (71%) F 22 (29%)	M 5 (100%) F 0	M 1507 (50.2%) F 1493 (49.8%)	M 8589 (50.7%) F 8340 (49.3%)
Creatinine [𝜇mol/]	Not available	40 [25–57]	72.5 [61–85]	Not available
eGFR [mL/min/1.73m^2^]	102 [93–111]	127 [98–151]	104.1 [91–116]	Not available

Virtual population cohorts were obtained by random resampling from the NHANES database. Continuous data are presented as median and interquartile range, proportions as absolute counts and percentages.

### 2.3 Clinical trial simulations: a prospective phase 2a study protocol in children 6– < 18 years of age

**Figure 2:**
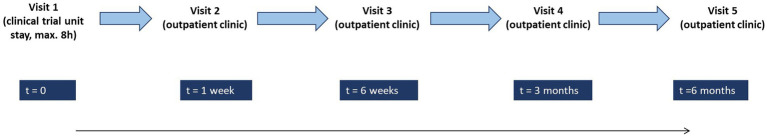
Flowchart depicting the key shared design elements (and pertaining constraints) of a prospective phase 2.a clinical study in children and adolescents with heart failure. Exploratory evaluation of the efficacy of empaglifozin in this population is based on a treatment period of at least 24 weeks, during which pharmacokinetics and pharmacodynamic markers should be characterised to justify the dose rationale and assess whether the observed exposure range correlates with clinical response.

A prospective trial has been proposed to investigate pharmacokinetics, ease of swallow, short-term safety and explore efficacy of empagliflozin among children 6 to < 18 years of age with heart failure, and Duchenne muscular dystrophy associated cardiomyopathy (ISRCTN12497973). This will be a prospective, open-label trial, in which children will take commercially available tablets of empagliflozin once daily for 6 months, with a full-day stay (max. 8 h) upon the first drug intake (Visit 1), and one opportunistic pharmacokinetic sample 1 week later (Visit 2). Further visits at 6 weeks (Visit 3), 3 (Visit 4) and 6 (Visit 5) months will assess the safety of empaglifozin and explore efficacy markers (Figure 2).

A preliminary assessment of the study feasibility indicates that recruitment has to be limited to a maximum of 12 participants. In addition, given the maximum blood volume allowed by current regulations (40, 41) and the need for safety monitoring blood draws, it will be possible to collect a maximum of 6 blood samples for pharmacokinetics at Visit 1, one opportunistic PK sample at Visit 2, and, potentially, one additional opportunistic PK sample at Visit 3. These restrictions represent an important risk, as both the dose rationale and eventual assessment of potential correlations between exposure and treatment response may not be established. In fact, there are various examples of inconclusive studies, which have relied on standard, empirical protocols, without any concern about the operating characteristics of the study design, other than a statistical power statement, which is often of limited value in exploratory studies, as outlined by the learning-confirming paradigm (42, 43). In a rare disease setting, the implementation of a clinical trial without a comprehensive evaluation of the informational content of a study protocol and its optimisation should be ethically questionable.

### 2.4 Sampling time and trial design optimisation

The $DESIGN feature in NONMEM^®^ was used to explore and optimise the proposed study design (44). This D-optimality based approach seeks to minimise the covariance of the parameter estimates, by assessing the Fisher information matrix and providing the expected model parameter precision and uncertainty (45, 46). The following constraints were set: *n* = 12 patients, maximum 6 samples on day 1, and 7 to 8 samples per patient over the whole trial, length of stay on day 1 of maximum 8 h, sampling time at Visit 2 (1 week after empagliflozin start) between 21 and 27 h after last drug intake. In addition, scenarios were assessed in which a single sampling schedule is used for all study participants, and individualised sampling schedule, or sampling matrices are evaluated. Scenarios with a total of 4, 5, and 6 samples were also explored (Supplementary Table 1). For the sampling matrices, two (i.e., 2 groups, 6 patients each) or four (i.e., 4 groups, 3 patients each) different sampling schedules have been considered. Moreover, alternative schedules were evaluated, including an additional opportunistic sample at Visit 3 (>3 weeks after empagliflozin start) in 50% or 100% of the participants, or to collect 8 samples in patients >20 kg body weight at Visit 1. Also, for comparison, parameter estimates and their precision were derived for a non-optimised schedule with 7 samples per patient at empirically chosen time points, for a rich sampling schedule with 12 participants contributing 13 samples each at empirically chosen time points (i.e., ethically not acceptable), and for a hypothetical study, albeit not feasible, with 40 participants contributing 13 samples each.

The D-optimisation step was implemented to identify potential designs in a more rapid and efficient manner than it would be achieved with traditional simulation re-estimation procedures. It also aimed at determining, for each of the explored sampling designs, the most informative sampling times. The impact of optimised designs on the accuracy and precision of the estimates was assessed by comparing the residual standard errors (%RSE) of the parameter estimates and the decrease in the objective function value (∆OFV).

### 2.5 Simulation re-estimation

The optimised sampling times identified in the previous step through the optimisation algorithm were used as input for four selected realistic scenarios (proposed sampling times were rounded to 5–15 min intervals according to clinical and practical considerations) with 6 samples at visit 1 based on a single sampling schedule for all participants, different sampling matrices with two or four groups as well as an individualised sampling schedule. Additionally, for comparison, we simulated a non-optimised, empirical sampling schedule; a rich sampling schedule with 13 samples; and a hypothetical design with 40 participants contributing 14 samples each (Table 2).

**Table 2:** Sampling scenarios implemented for protocol optimisation using the simulation re-estimation (SSE) procedures.

Scenario	Description	# of participants	Schedule	Rounded sampling times (hours)
1	Hypothetical study with rich sampling	12	Visit 1: 12 samples Visit 2: 1 sample Visit 3: 1 sample	0.5, 1.0, 1.5, 2.0, 2.5, 3.0, 3.5, 4, 5, 6, 7, 8, 168, 507
2	*Ibid.*	40	*Ibid.*	*Ibid.*
3	Empirical, non-optimised sampling scheme	12	Visit 1 (max. 8 h observation): 6 samples Visit 2: 1 sample	0.5, 1, 2, 4, 6, 8, 168
4	Optimised sampling scheme, 1 group	12	Visit 1 (max. 8 h observation): 6 samples Visit 2: 1 sample	1.0, 2.2, 2.5, 4.4, 7.7, 8.0, 168
5	Optimised sampling scheme, 2 groups	12	Visit 1 (max. 8 h observation): 6 samples Visit 2: 1 sample Visit 3: 1 sample in 50% of participants	Sampling group 1: 1.0, 2.3, 2.6, 4.0, 7.9, 8.0, 168, 507 Sampling group 2: 1.0, 2.4, 2.5, 4.0, 7.9, 8.0, 168
6	Optimised sampling scheme, 4 groups	12	Visit 1 (max. 8 h observation): 6 samples Visit 2: 1 sample Visit 3: 1 sample in 50% of participants	Sampling group 1: 0.7, 0.9, 1.9, 2, 4, 8, 168 Sampling group 2: 0.5, 1, 2.5, 4, 7.8, 8, 168, 507 Sampling group 3: 0.7, 1, 2.1, 4, 7.7, 8, 168 Sampling group 4: 0.7, 1, 2.2, 4, 7.6, 8, 168, 507
6.a	Optimised sampling scheme, 4 groups	8	*Ibid.*	*Ibid.*
6.b	Optimised sampling scheme, 4 groups, max. 6 h at visit 1	12	Visit 1 (max. 6 h observation): 6 samples Visit 2: 1 sample Visit 3: 1 sample in 50% of participants	Sampling group 1: 0.6, 0.9, 1.6, 5.1, 5.9, 6, 168 Sampling group 2: 0.5, 1, 2, 2.2, 5.9, 6, 168, 507 Sampling group 3: 1, 1.7, 2.4, 2.6, 5.9, 6, 168 Sampling group 4: 1, 2.1, 2.6, 4.3, 5.9, 6, 168, 502
6.c	Optimised sampling scheme, 4 groups, only Visit 2 for all	12	Visit 1 (max. 8 h observation): 6 samples Visit 2: 1 sample (in all participants)	Sampling group 1: 0.5, 1, 2.3, 4.1, 7.7, 8, 168 Sampling group 2: 1, 2.5, 2.6, 4.2, 7.7, 8, 168 Sampling group 3: 1, 1.1, 2.4, 4.2, 7.9, 8, 168 Sampling group 4: 0.7, 0.9, 2.3, 3.8, 7.5, 7.9, 168
6.d	Optimised sampling scheme, 4 groups, Visit 3 for all	12	Visit 1 (max. 8 h observation): 6 samples Visit 2: 1 sample (in all participants) Visit 3: 1 sample (in all participants)	Sampling group 1: 0.5, 1, 1.9, 3.9, 7.7, 8, 168, 507 Sampling group 2: 1, 1.1, 2.2, 2.3, 4, 8, 168, 502 Sampling group 3: 0.7, 0.9, 2.1, 4, 7.4, 8, 168, 502 Sampling group 4: 0.7, 0.9, 2, 4, 7, 7.9, 168, 501
6.e	Optimised sampling scheme, 4 groups, only 5 samples at Visit 1, Visits 2 and 3 for all participants	12	Visit 1 (max. 8 h observation): 5 samples Visit 2: 1 sample Visit 3: 1 sample (in all participants)	Sampling group 1: 0.9, 2.2, 2.3, 7.9, 8, 165. 507 Sampling group 2: 0.9, 2.4, 2.5, 7.9, 8, 165, 507 Sampling group 3: 1, 2.4, 2.5, 8, 8, 168, 501 Sampling group 4: 1, 2.4, 2.5, 7.9, 8, 165, 507
7	Optimised sampling scheme, individualised (i.e., 12 different sampling schedules)	12	Visit 1 (max. 8 h observation): 6 samples Visit 2: 1 sample Visit 3: 1 sample (in 50% of participants)	See Supplementary Table S3.

Given the rarity of the condition, informative and non-informative priors were used to support parameter estimation in the simulation re-estimation step, which included 500 trial replicates. The ratio between re-estimated and originally simulated pharmacokinetic parameter was calculated for each primary and secondary pharmacokinetic parameter of interest. A well-performing sampling scheme is expected to produce estimates close to 1.0 and with a relatively narrow distribution (i.e., 0.7–1.3). Whilst there is no specific range for defining accuracy, the proposed range takes into account the potential implications of varying exposure for the underlying pharmacological effect.

The selected sampling schedules were further evaluated based on a smaller sample size (i.e., 4, 6, 8 or 10 patients). Moreover, additional variants of this scenario were explored (patients staying only a maximum of 6 h at Visit 1, obtaining an opportunistic sample at Visit 3 from all or none of the participants, limiting the samples at Visit 1 to a maximum of 5, but obtaining an opportunistic sample at Visit 3 from all participants).

### 2.6 Summary statistics

While summarising literature findings, weighted means were calculated. If needed, means and standard deviations were transformed into median and interquartile range by simulating a large normal sample distribution and calculating the pertaining summative statistics.

For the optimisation steps, the relative precision of secondary pharmacokinetic parameters was used as metrics for optimisation. Parameters of interest included the area under the concentration vs. time curve (AUC), the peak concentration (C_max_), the time at which peak concentrations occur (T_max_), and average steady-state concentration (C_ss_). AUC and C_max_ were simulated for a virtual paediatric cohort of patients across a weight range between 10 and 90 kg, following administration of two dose levels (10 and 25 mg), which reflect the currently commercially available tablets of empagliflozin. Sampling times were handled as discrete data, where appropriate. Descriptive, summary statistics for continuous data included median and interquartile range, or mean and standard deviation. A two-sided *t*-test was used, with *p*-values of <0.05 as threshold for statistical significance.

Data handling, graphical and tabular summaries were performed in R statistics (v.4.2.2) with the support of R studio (2022.07.2 or higher) (47). Modelling and simulation steps were implemented in NONMEM^®^ v.7.5 (ICON Development Solutions, Ellicott City, United States), in combination with PsN^®^-Toolkit (v. 5.3.0) and Pirana^®^ v.2.9.9.

## 3 Results

### 3.1 Systematic review

The literature search process is summarised in Supplementary Figure 1. We identified *n* = 6 articles reporting two population pharmacokinetic models (34–39). Each of these was then replicated and/or adapted in slightly different populations, as summarised in Supplementary Table 2. They included 5,252 patients, mean 57.6 ± 10.9 years of age with type 1 (*n* = 1,316) or type 2 (*n* = 3,936) diabetes mellitus. Investigated doses ranged from 1 to 100 mg, from single dose up to 12 weeks once daily administration. All studies were performed using nonlinear mixed effects modelling, as implemented in NONMEM^®^, four of them performed a bootstrap analysis and only two articles reported visual predictive checks. Five studies reported values for the primary and secondary pharmacokinetic parameters (35–39).

The selected model (which included details on the parameter estimates and evidence of acceptable predictive performance) was built on data from 56 type 1 diabetes mellitus patients who provided 1814 blood samples. These patients had a mean (sd) age of 41 ± 11 years, weight of 79.3 ± 14.3 kg, with normal renal function (36). Patients were given empagliflozin doses of 2.5 mg (*n* = 19), 10 mg (*n* = 19) or 25 mg (*n* = 18) once daily for 28 days. The model describes the pharmacokinetics of empagliflozin with 2-compartments and sequential zero- and first-order absorption, an absorption lag time to the central compartment, and a first-order elimination. Random effect parameters were identified for apparent clearance (CL/F), peripheral volume of distribution (V_p_/F), intercompartmental clearance (Q/F), duration of the zero-order absorption (D1), absorption rate constant (k_a_) and absorption lag time (ALAG1). Residual variability was described by a proportional error model. Body mass index was identified as a covariate explaining the interindividual variability of CL/F, V_c_/F, V_p_/F, and Q/F.

### 3.2 Scaling of pharmacokinetics and extrapolation from adults to paediatric patients

Prior to evaluating the effect of body weight on the disposition of empagliflozin, model performance was assessed by replicating the reported results in adults based on a VPC stratified by dose level (2.5, 10 mg, and 25 mg) (Supplementary Figure 2) (36). A similar assessment was performed after model reparameterisation according to allometric principles (Supplementary Figure 3).

**Figure 3:**
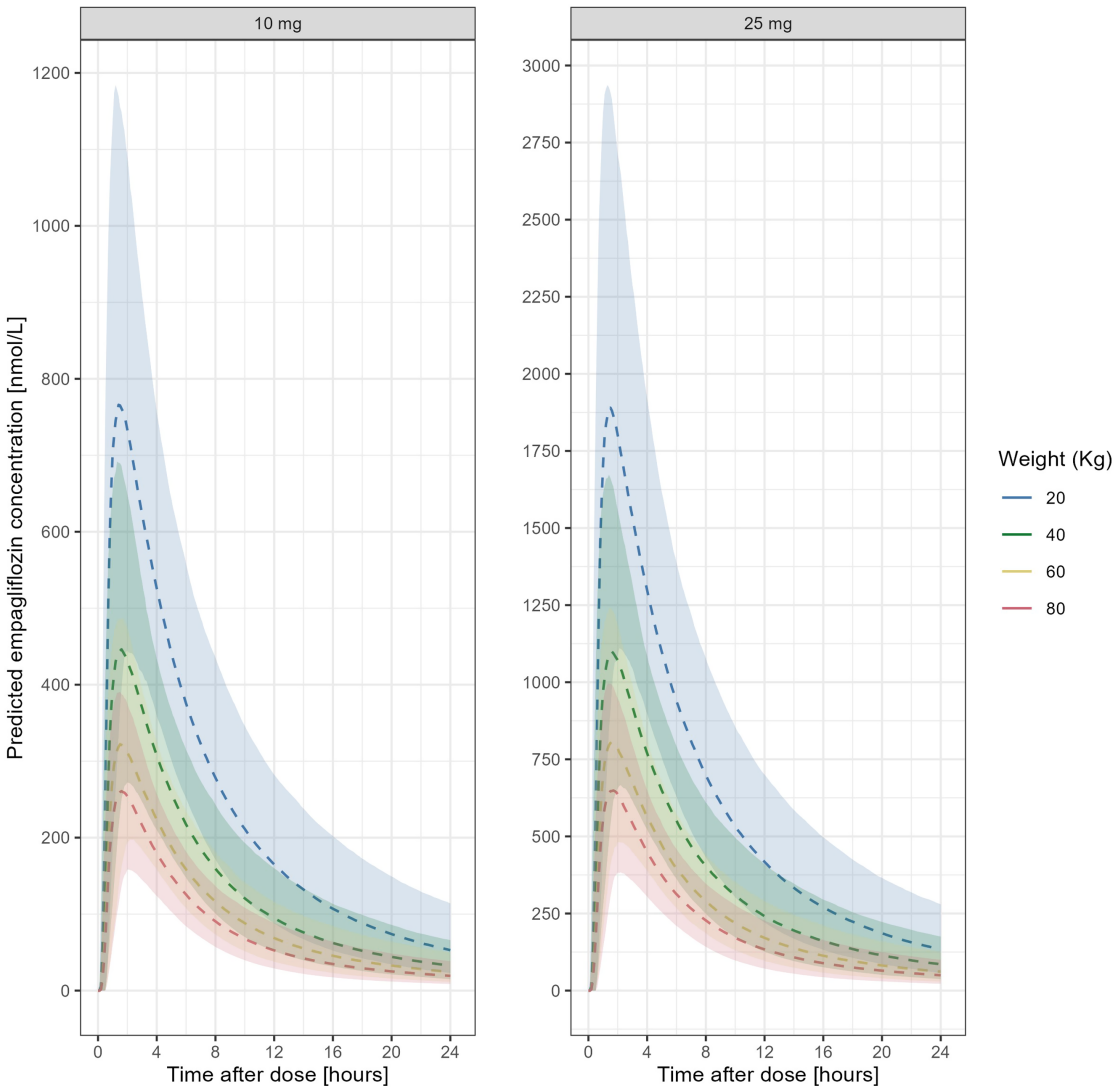
Predicted empaglifozin concentration vs.time profile in individuals of 20, 40, 60 or 80 kg after administration of doses of 10 mg or 25 mg empagliflozin. Dashed lines represent the predicted median concentration, while the shaded areas depict the 95%-prediction intervals, as assessed by the model.

The model including allometrically scaled parameters was subsequently used to simulate individual concentration vs time profiles in a virtual paediatric population (Figure 3) and support the selection of doses that ensure empagliflozin exposure that corresponds to the observed range in adults, or specifically to an exposure range that is ≥70% of the AUC obtained in normal weight adults with the standard 10 mg once daily regimen (i.e., recommended for adults with heart failure). Similar criteria were used to ensure safe exposure, defined as ≤ 130% of the AUC and C_max_ obtained in adults receiving the maximum recommended dose of 25 mg once daily (i.e., recommended, when needed, for adults with type 2 diabetes mellitus).

**Figure 4:**
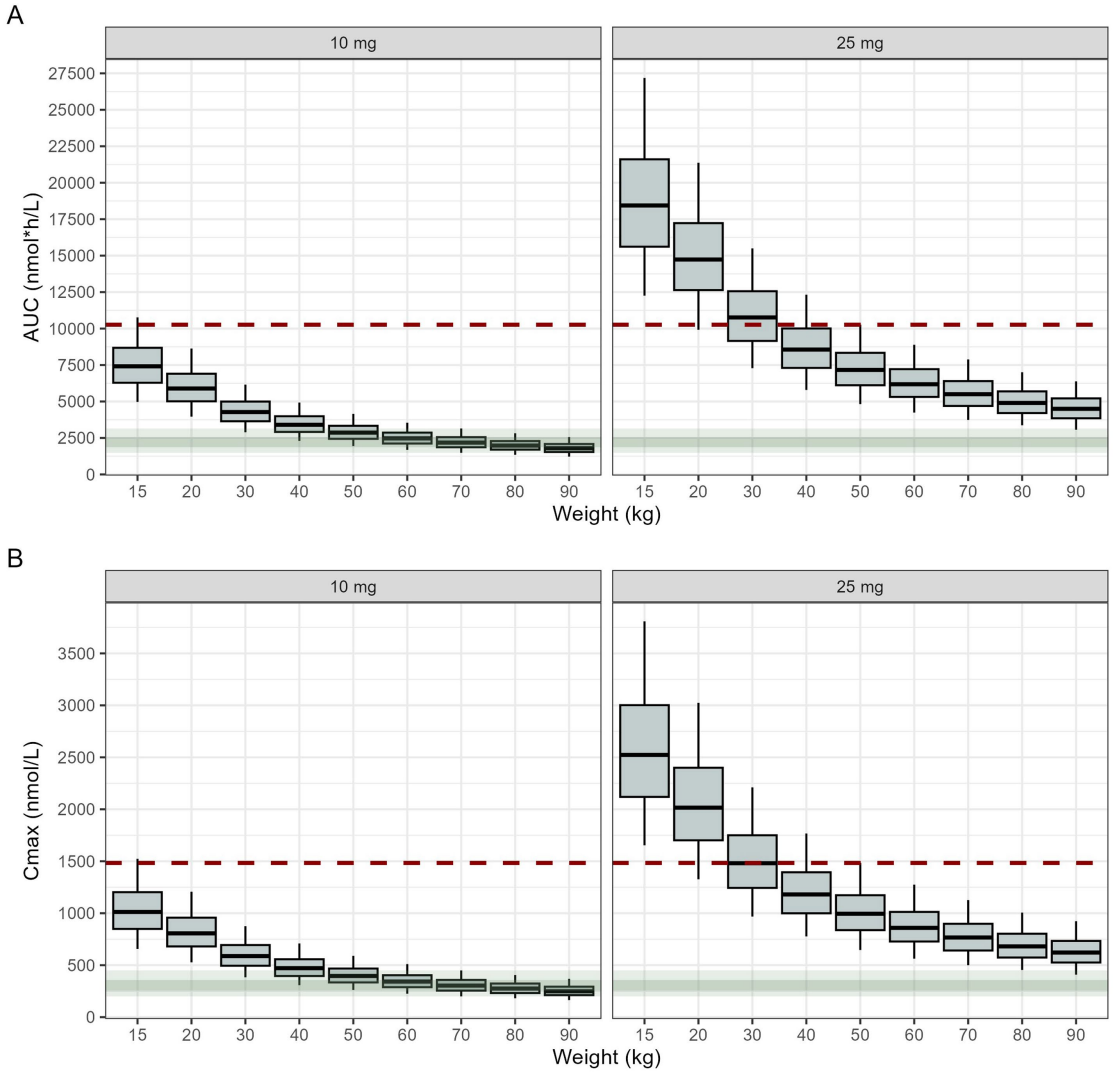
Model-predicted area under the concentration vs time curve, AUC_0–24_ **(A)**, and maximal concentration, C_max_ **(B)** after administration of once daily doses of 10 (left) or 25 mg (right) empagliflozin. Results are stratified by body weight and depict a clinically relevant range for the paediatric population affected by heart failure. Box and whiskers depict median and interquartile range, while bars represent the 5th–95th percentile range. The shaded areas (dark green 25th–75th percentiles, light green 5th to 95th percentiles) represent the model-predicted exposure in a 70 kg adult after administration of once daily doses of 10 mg empagliflozin (recommended dose for an adult with heart failure, i.e., reference exposure for extrapolation of efficacy). The red dashed line depicts the 95th percentile of the model-predicted exposure in a 50 kg adult receiving once daily doses of 25 mg empagliflozin (maximal recommended adult standard dose, reference exposure range for the extrapolation of safety).

Whilst these thresholds may be somewhat arbitrary, they take into account known unexplained variability in drug disposition and formulation-related variation in exposure. Simulated AUC and C_max_ for virtual paediatric patients across a wide range of body weights (10, 15, 20, 30, 40, 50, 60, 70, 80 or 90 kg) are shown in Figure 4. From these results, it becomes evident that children below 15 kg should not be included in the study, because they would exceed the upper limit of exposure, as determined by the reference of 25 mg once daily in adults with type 2 diabetes mellitus. Median empagliflozin exposure ratio between adults and children ≥15 kg was within the target range. This suggests that the proposed regimen is expected to be safe, with the 75th percentiles of the exposure ratio remaining at <1.3 for both AUC and C_max_ (Table 3).

**Table 3:** Predicted secondary pharmacokinetic parameters after administration of 10 and 25 mg daily doses of empagliflozin, to paediatric patients with 15 kg body weight, as compared to adult subjects with 50 kg and 70 kg body weight. The ratio of these parameters provides the basis for the evaluation of the proportion of paediatric patients who may exceed the exposure range in adults receiving the highest recommended dose of empaglifozin (i.e., 25 mg).

	C_max_	AUC	t_max_	C_ss_
70 kg, 25 mg	766.5 [641.3–898.3]	5,499 [4,696–6,398]	1.4 [1.2–1.8]	229.1 [195.7–266.6]
70 kg, 10 mg	303.6 [255.8–357.8]	2,182 [1,864–2,552]	1.4 [1.2–1.7]	90.9 [77.7–106.4]
50 kg, 25 mg	995.1 [837.5–1,173.2]	7,163 [6,115–8,338]	1.4 [1.2–1.7]	298.4 [254.8–347.4]
50 kg, 10 mg	396.2 [334.2–467.9]	2,865 [2,439–3,332]	1.4 [1.2–1.7]	119.4 [101.6–138.8]
15 kg, 25 mg	2,523 [2,119.4–3,002.1]	18,438 [15,614–21,605]	1.2 [1.0–1.6]	768.2 [650.6–900.2]
15 kg, 10 mg	1,012 [849.0–1,202.5]	7,412 [6,285–8,682]	1.3 [1.0–1.6]	308.9 [261.9–361.7]
Ratio 15 kg 10 mg/50 kg 25 mg	1.02 [0.80–1.30]	1.03 [0.82–1.30]	NA	1.03 [0.82–1.30]
Ratio 15 kg 10 mg/70 kg 25 mg	1.33 [1.04–1.69]	1.35 [1.08–1.68]	NA	1.35 [1.08–1.68]

The green-shadowed row represents a typical 70 kg adult patient with heart failure receiving a dose of 10 mg once daily (i.e., the recommended dose for heart failure), the red-shaded row depicts a low-weight adult of 50 kg receiving the maximal recommended adult dose of 25 mg once daily, which was used to calculate a putative “safety” margin (blue-shaded row) for the lowest weight inclusion criterion (i.e., 15 kg) in a prospective paediatric study. Continuous data are presented as median and interquartile range.

### 3.3 Sampling time optimisation

Given that only body weight was identified as a significant covariate, we generated a large virtual cohort with normally distributed weights, with mean (sd) and distribution (38.1 ± 16.8 kg) reflecting the actual clinical population characteristics. Random sampling was then used to optimise sampling procedures, assuming study protocols with 12, 18 and 40 patients. Decrease in the objective function value (∆OFV), and precision of the estimates were similar across the different scenarios (Supplementary Table 3), with only the scenarios with 3 and 4 samples (Optima 7 and 8) showing smaller ∆OFV and higher RSE. However, the precision of the interindividual variability parameters was poorly estimated. A hypothetically large study including 40 patients was required to allow adequate estimates of interindividual variability in clearance and volume of distribution. Yet, this remained tricky for intercompartmental clearance, given the trial constraints (max. 8 h at Visit 1, limited number of samples) (Supplementary Table 3).

The optimised sampling times to carry forward in the simulation re-estimation step were rounded taking into account feasibility criteria (Table 2). The optimisation algorithm was re-run, closely mirroring the original results (data not shown).

### 3.4 Clinical trial simulations

**Figure 5:**
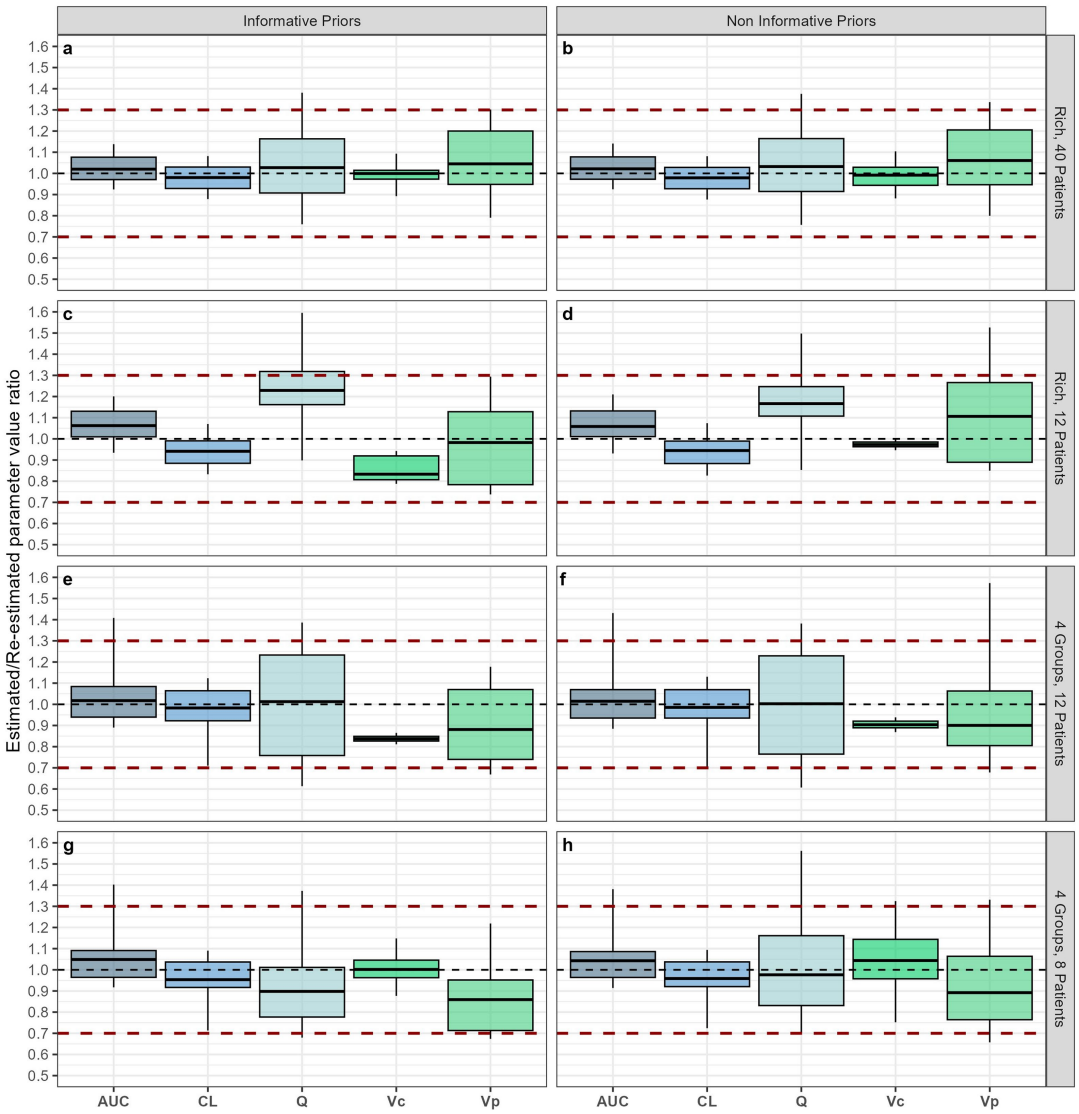
Estimates of primary and secondary pharmacokinetic parameters after simulation and re-estimation of 500 trials with 8, 12, or 40 subjects each, according to the predefined simulation scenarios with varying sampling schedules. The first two rows depict ideal, but unfeasible, rich sampling scenarios (panels 5.a to 5.d). The third and fourth rows depict selected sampling scenarios (4 sampling groups, optimised sampling times) with either 12 (panels 5.e and 5.f) or 8 (panels 5.g and 5.h) participants. A sampling scheme was deemed well-performing if the ratio between the “true” and the “re-estimated” parameter values were close to 1.0 with a relatively narrow distribution (i.e., 0.7–1.3). CL, clearance; V_c_, central volume of distribution; V_p_, peripheral volume of distribution; Q, intercompartmental clearance; AUC, area under the concentration-time curve.

Twelve scenarios were simulated (Table 2; Supplementary Table 4). Interestingly, the empirical, rich sampling schedule including 12 samples on Day 1, and one opportunistic sample at Visit 2 and 3, did not perform very well. The ratio of re-estimated versus simulated parameter estimates included 1.0 in its interquartile range only for the peripheral volume of distribution, and with a pretty large spread. All the remaining pharmacokinetic parameters (CL, Q and AUC), whilst still showing a median within the pre-set acceptability range of 0.7–1.3, did not include 1.0 within their interquartile range (Figures 5c,d). The precision clearly improves with a hypothetical trial including 40 patients, each contributing with 14 samples (Figures 5a,b). However, these conditions are neither realistically feasible nor ethically allowed. On the other hand, the optimised sampling scheme with 12 patients divided into 4 different sampling groups, with both informative (Figure 5e) and, even more so, non-informative priors (Figure 5f), delivered better estimates than the empirical design with rich sampling. Of note is that only the central volume of distribution did not include 1.0 within its interquartile range [0.90 (IQR 0.89–0.92)]. The empirical, non-optimised sampling schedule with 7 samples (Supplementary Figures 4a,b), the optimised sampling schedule with 1 (Supplementary Figures 4c,d) and 2 sampling groups (Supplementary Figures 4e,f), and the individually optimised sampling schedule (i.e., one for each patient) showed lower performance (Supplementary Figures 4g,h). Specifically, these sampling matrices were clearly inferior to the selected sampling matrix with respect to central and peripheral volumes of distribution, with V_c_ falling outside the pre-defined 0.7–1.3 acceptability range (Supplementary Figure 4).

The optimised sampling schedule with 4 different sampling groups performed well also when considering the inclusion of just 8 patients instead of 12 (Figures 5g,h), whilst the reduction in sample size had a significant effect on the precision of estimates when a study including only 6 (two groups of 3 patients) or 4 patients (1 patient per sampling group) were considered.

Lastly, we explored the impact of not collecting any opportunistic sample at Visit 3 (Supplementary Figures 5a,b), performing it in all participants (Supplementary Figures 5c,d), performing it in all participants while limiting the samples at Visit 1 to five (Supplementary Figures 5e,f), as well as the possibility of limiting the hospital stay at Visit 1 to a maximum of 6 h (Supplementary Figures 5g,h). All these scenarios performed less well than the selected scenario with 4 sampling schedules (Figures 5e,f).

## 4 Discussion

This study shows that clinical protocols aimed at evaluating repurposed drugs in rare paediatric diseases can and should be optimised. It is imperative that data generation in proof of concept studies in such vulnerable patients is efficient, accurate and relevant, and a dose rationale defined as early as possible (7, 9). Whilst the proposed prospective protocol for a phase 2a study relies on the assumption of comparable disease processes and similar concentration-effect relationship in adults and children, the approach presented here can be applied to a wider range of conditions, including those in which differences in the underlying PKPD relationships are anticipated between populations.

It is worth highlighting that we have undertaken a comprehensive review of the literature to ensure the robustness of the extrapolation step, including careful evaluation of the population pharmacokinetic model performance in adults prior to its reparameterisation to incorporate an allometric function describing weight-related changes in the disposition of empagliflozin. Thanks to the scaling of pharmacokinetics, we were able to identify the lowest weight of 15 kg, which will allow paediatric patients to be exposed to commercially available tablets without exceeding the upper, safe exposure range, as determined by the reference of 25 mg once daily in adults. This posology is currently recommended, when improved glycaemic control is required in adults with type 2 diabetes mellitus. This threshold is based on extensive data showing good tolerability in adults. However, we acknowledge that lower-weight children might be exposed to empagliflozin levels that are higher than required. This study specifically serves to define safe inclusion criteria for a prospective trial using commercially available tablets. Notably, results from the planned proof-of-concept will form the basis for further recommendations for subsequent clinical trials. Our focus is to primarily estimate population typical values of the pharmacokinetic parameters. Interindividual variability and covariates explaining it will be characterised in larger late phase studies.

Even though concerns may be raised about the operating characteristics of a protocol based on a limited sample size, there is plenty of evidence showing the advantages of a model-based approach for the analysis of pharmacokinetic, pharmacodynamic, efficacy and safety data (48, 49). We have screened and compared several designs, exploring different sampling matrices, visit intervals, and group sizes. In doing so, we assessed the robustness and reliability of the proposed design using two optimisation methods (50, 51). At the end, the scenario with 4 sampling groups provided the best estimates for both primary and secondary PK parameters, with ratios very close to 1 and interquartiles within the 70–130% range.

We were initially surprised how little both the objective function and the residual standard errors (RSE) varied across the screened scenarios. However, it became clear that, while the estimation of the PK parameters was acceptable (with RSE < 30% for most scenarios), the estimation of the interindividual variability was more challenging, particularly for Q and V_3_. This was not unexpected, given the relatively small sample size. In fact, the only way to improve the precision of parameter estimates associated with interindividual variability was to increase the number of participants (Supplementary Table 3), which is not realistically feasible in the proposed prospective phase 2a study (ISRCTN12497973). Consequently, pharmacokinetic data will still have to be collected as part of subsequent phase 2b/phase 3 studies.

Across the different scenarios, the optimised sampling times proposed by the optimisation algorithm (Table 2) made pharmacological sense, and tended to cluster around key moments of empagliflozin disposition (Figure 3), with a first cluster at around 0.5–2 h (primarily related to the absorption process), a second and a third cluster at around 4 h and 7–8 h, respectively, which reflect the elimination process. It was interesting to note that at Visit 2 and 3, the optimisation algorithm sought a balance between samples characterising elimination (sampling just before drug administration) and absorption (sampling just after drug administration).

We might also be able to reduce the sample size to 8 patients, without significant loss of precision (Figures 5g,h). However, one needs to consider that safety and efficacy markers will also be evaluated in the prospective trial, so that inclusion of 12 patients remains preferable. Indeed, already with 12 patients the trial will be pretty limited in its ability to detect adverse events (Supplementary Table 5). Nevertheless, such evidence is relevant for the trial steering committee, sponsor and regulatory authorities. In case difficulties in recruitment will ensue, the current investigation provides convincing data that the primary objective may be achieved even with only 8 participants.

From a technical perspective, we have also shown how prior knowledge can be used in conjunction with extrapolation principles. Informative and non-informative priors generated similar results. Despite the lack of significant differences, we opted for the use of non-informative priors, as extrapolation of pharmacokinetics was based on data from a different population (adults) and disease (diabetes mellitus). It is also worth mentioning that the $DESIGN function in NONMEM^®^ is relatively recent (versions 7.5.0 and following). Hence, we believe that our experience with this feature deserves further discussion. In a setting with strong constraints and small sample size, optimisation procedures and operating characteristics of a protocol should be aligned to maintain the oversight of the clinical aims. In this case, our overarching aim was to establish the dose rationale and investigate the impact of body weight on exposure. Consequently, optimisation is focused on CL and AUC. This implies their prioritisation over the remaining parameters (i.e., V_p_ and Q, V_c_ and C_max_). Indeed, it has been shown that efficacy of SGLT2 inhibitors is associated with the overall exposure, as measured by AUC or steady-state concentrations (35, 37, 39).

From a clinical perspective, this study shows how one can mitigate ethical and practical challenges, whilst ensuring the scientific robustness of the data collected in a clinical trial in children. In the context of rare diseases, every piece of information, and each included participant, becomes even more important, in both relative and absolute terms, than in a traditional trial performed in adults (27, 52, 53). As opposed to entrenched beliefs, empirical protocols are less informative than optimised ones. The impact of optimisation procedures represents more than just a statistical improvement. Generation of data with higher precision affects the overall quality of a study, including lower burden to participants, improved feasibility and recruitment (54). These features also have an impact on costs and time, shortening the time required for evidence generation (55). This is critical, because it accelerates the pathway towards therapy availability in clinical care. In the context of rare diseases, further improvement may be achieved by expanded use of a Bayesian framework, which has evolved to a wider range of approaches, such as dynamic borrowing, allowing for more efficient integration of information from external sources (56, 57).

We also acknowledge the limitations in the study. Unfortunately, there are no population pharmacokinetics models of empagliflozin in adults with heart failure available. Therefore, we extrapolated information from adults with diabetes mellitus to children with heart failure. The extrapolation across disease conditions is based on the absence of evidence suggesting major disease-related changes in drug disposition. A recent comparison of through concentrations (C_through_) from trials on adults with type 2 diabetes versus heart failure showed higher C_through_ in heart failure, which was partially explained by weight and renal function at baseline (58). This difference highlights the need for specific pharmacokinetic studies in heart failure. At the same time, however, the anticipated effect size, particularly in patients with preserved renal function, allows confidence in the use of extrapolation for study design optimisation. Importantly, no major differences in adults versus adolescents with type 2 diabetes mellitus were noticed (18, 59, 60). Moreover, for dapagliflozin, another SGLT2-inhibitor very similar to empagliflozin, in adults it has been demonstrated that pharmacokinetics is similar between heart failure with reduced ejection fraction and patients with type 2 diabetes mellitus (61). Second, we also recognise that other clinical factors than body weight may affect the disposition of empagliflozin. As such, the impact of ontogeny and maturation processes was deemed negligible across the studied age range. Indeed, the primary route of metabolism of empagliflozin is glucuronidation by uridine 5′ diphosphoglucuronosyltransferases (5’-UGT) 1A3, 1A8, 1A9, and 2B7. 5’-UGT expression and activity increases markedly during the first weeks of life, while it is unclear whether, how much and how long maturation extends beyond the age of 2 years (24, 62–64). Third, model misspecification is a risk that cannot be excluded. Indeed, the planned prospective trial will allow us to validate and refine the current pharmacokinetic model to inform the dose rationale for subsequent efficacy trials.

## 5 Conclusion

We have shown how modelling, simulation and extrapolation concepts can be integrated with optimality methodology to inform the design of a prospective phase 2a study in children affected by heart failure, a rare condition for which limited options are available. Whilst our approach aims to enhance the efficiency and quality of the data generated in support of drug repurposing, the implementation of *in silico* simulation scenarios allowed us to minimise patient burden, improving study feasibility. More specifically, we have shown that the use of four sampling schedules with 12 participants, each contributing with 6 samples at Visit 1, one opportunistic sample at Visit 2 and, for half of the participants, an opportunistic sample at Visit 3, provides the most informative trial design. Moreover, our study illustrates the implementation of the principles outlined in the recent update of the ICH guideline on paediatric extrapolation (26). Paediatric heart failure represents a typical situation where randomisation of paediatric patients to subtherapeutic doses, or use of placebo, may be unethical, and/or available safety data may not support evaluation of higher doses/exposures. In these circumstances, dose selection based on exposure matching is reasonable and pragmatic and is predicated on the expectation that a comparable response at the target drug exposure is likely to be achieved.

## Data Availability

Publicly available datasets were analysed in this study. Data and model codes can be requested.

## References

[1] Rossano JW Kim JJ Decker JA Price JF Zafar F Graves DE . Prevalence, morbidity, and mortality of heart failure-related hospitalizations in children in the United States: a population-based study. J Card Fail. (2012) 18:459–70. doi: 10.1016/j.cardfail.2012.03.001, 22633303

[2] Newland DM Law YM Albers EL Friedland-Little JM Ahmed H Kemna MS . Early clinical experience with dapagliflozin in children with heart failure. Pediatr Cardiol. (2023) 44:146–52. doi: 10.1007/s00246-022-02983-0, 35948644

[3] Andrews RE Fenton MJ Dominguez T Burch M British Congenital Cardiac Association. Heart failure from heart muscle disease in childhood: a 5-10 year follow-up study in the UK and Ireland. ESC Heart Fail. (2016) 3:107–14. doi: 10.1002/ehf2.12082, 27812385 PMC5066798

[4] Towbin JA Lowe AM Colan SD Sleeper LA Orav EJ Clunie S . Incidence, causes, and outcomes of dilated cardiomyopathy in children. JAMA. (2006) 296:1867–76. doi: 10.1001/jama.296.15.1867, 17047217

[5] Hollander SA Nandi D Bansal N Godown J Zafar F Rosenthal DN . A coordinated approach to improving pediatric heart transplant waitlist outcomes: a summary of the ACTION November 2019 waitlist outcomes committee meeting. Pediatr Transplant. (2020) 24:e13862. doi: 10.1111/petr.13862, 32985785

[6] D'Addese L Joong A Burch M Pahl E. Pediatric heart transplantation in the current era. Curr Opin Pediatr. (2019) 31:583–91. doi: 10.1097/MOP.0000000000000805, 31335745

[7] Clout AE Della Pasqua O Hanna MG Orlu M Pitceathly RDS. Drug repurposing in neurological diseases: an integrated approach to reduce trial and error. J Neurol Neurosurg Psychiatry. (2019) 90:1270–5. doi: 10.1136/jnnp-2019-320879, 31171583

[8] Simeoli R Lava SAG Di Deo A Roversi M Cairoli S Tambucci R . Pharmacokinetic evaluation of oral viscous budesonide in paediatric patients with eosinophilic oesophagitis in repaired oesophageal atresia. Pharmaceutics. (2024) 16:872. doi: 10.3390/pharmaceutics16070872, 39065569 PMC11280286

[9] Romano F D'Agate S Della Pasqua O. Model-informed repurposing of medicines for SARS-CoV-2: extrapolation of antiviral activity and dose rationale for paediatric patients. Pharmaceutics. (2021) 13:1299. doi: 10.3390/pharmaceutics13081299, 34452260 PMC8399437

[10] Healy P Verrest L Felisi M Ceci A Della Pasqua O GAPP Consortium. Dose rationale for gabapentin and tramadol in pediatric patients with chronic pain. Pharmacol Res Perspect. (2023) 11:e01138. doi: 10.1002/prp2.1138, 37803937 PMC10558965

[11] Loss KL Shaddy RE Kantor PF. Recent and upcoming drug therapies for pediatric heart failure. Front Pediatr. (2021) 9:681224. doi: 10.3389/fped.2021.681224, 34858897 PMC8632454

[12] McMurray JJV Solomon SD Inzucchi SE Køber L Kosiborod MN Martinez FA . Dapagliflozin in patients with heart failure and reduced ejection fraction. N Engl J Med. (2019) 381:1995–2008. doi: 10.1056/NEJMoa1911303, 31535829

[13] Packer M Anker SD Butler J Filippatos G Pocock SJ Carson P . Cardiovascular and renal outcomes with empagliflozin in heart failure. N Engl J Med. (2020) 383:1413–24. doi: 10.1056/NEJMoa2022190, 32865377

[14] McDonagh TA Metra M Adamo M Gardner RS Baumbach A Böhm M . 2021 ESC guidelines for the diagnosis and treatment of acute and chronic heart failure: developed by the task force for the diagnosis and treatment of acute and chronic heart failure of the European Society of Cardiology (ESC). With the special contribution of the heart failure association (HFA) of the ESC. Eur J Heart Fail. (2022) 24:4–131. doi: 10.1002/ejhf.2333 35083827

[15] Heidenreich PA Bozkurt B Aguilar D Allen LA Byun JJ Colvin MM . 2022 AHA/ACC/HFSA guideline for the Management of Heart Failure: executive summary: a report of the American College of Cardiology/American Heart Association joint committee on clinical practice guidelines. J Am Coll Cardiol. (2022) 79:1757–80. doi: 10.1016/j.jacc.2021.12.011, 35379504

[16] Braunwald E. Gliflozins in the management of cardiovascular disease. N Engl J Med. (2022) 386:2024–34. doi: 10.1056/NEJMra2115011, 35613023

[17] Zelniker TA Braunwald E. Mechanisms of cardiorenal effects of sodium-glucose cotransporter 2 inhibitors: JACC state-of-the-art review. J Am Coll Cardiol. (2020) 75:422–34. doi: 10.1016/j.jacc.2019.11.031, 32000955

[18] Lava SAG Laurence C Di Deo A Sekarski N Burch M Della Pasqua O. Dapagliflozin and empagliflozin in paediatric indications: a systematic review. Paediatr Drugs. (2024) 26:229–43. doi: 10.1007/s40272-024-00623-z, 38635113

[19] Lava SAG. SGLT2 inhibitors for paediatric heart failure: time for innovative trials. Int J Cardiol. (2025) 438:133521. doi: 10.1016/j.ijcard.2025.133521, 40544878

[20] Nagendra L Dutta D Girijashankar HB Khandelwal D Lathia T Sharma M. Safety and tolerability of sodium-glucose cotransporter-2 inhibitors in children and young adults: a systematic review and meta-analysis. Ann Pediatr Endocrinol Metab. (2024) 29:82–9. doi: 10.6065/apem.2346162.081, 38163851 PMC11076228

[21] Benjamin DK Jr Smith PB Jadhav P Gobburu JV Murphy MD Hasselblad V . Pediatric antihypertensive trial failures: analysis of end points and dose range. Hypertension. (2008) 51:834–40. doi: 10.1161/HYPERTENSIONAHA.107.108886, 18332283 PMC2782749

[22] Cella M Knibbe C Danhof M Della Pasqua O. What is the right dose for children? Br J Clin Pharmacol. (2010) 70:597–603. doi: 10.1111/j.1365-2125.2009.03591.x, 21087295 PMC2950994

[23] Shaddy RE Boucek MM Hsu DT Boucek RJ Canter CE Mahony L . Carvedilol for children and adolescents with heart failure: a randomized controlled trial. JAMA. (2007) 298:1171–9. doi: 10.1001/jama.298.10.1171, 17848651

[24] Bellanti F Della Pasqua O. Modelling and simulation as research tools in paediatric drug development. Eur J Clin Pharmacol. (2011) 67:75–86. doi: 10.1007/s00228-010-0974-3, 21246352 PMC3082698

[25] Vermeulen E van den Anker JN Della Pasqua O Hoppu K van der Lee JH. Global research in Paediatrics (GRiP). How to optimise drug study design: pharmacokinetics and pharmacodynamics studies introduced to paediatricians. J Pharm Pharmacol. (2017) 69:439–47. doi: 10.1111/jphp.12637, 27671925 PMC6084327

[26] International Council for Harmonisation of Technical Requirements for Pharmaceuticals for Human Use ICH E11A Guideline on pediatric extrapolation August 2024. Available online at: https://www.ema.europa.eu/en/documents/scientific-guideline/ich-guideline-e11a-pediatric-extrapolation-step-5_en.pdf (Accessed November 1, 2024).

[27] Bellanti F Di Iorio VL Danhof M Della Pasqua O. Sampling optimization in pharmacokinetic bridging studies: example of the use of deferiprone in children with β-thalassemia. J Clin Pharmacol. (2016) 56:1094–103. doi: 10.1002/jcph.708, 26785826

[28] Borella E Oosterholt S Magni P Della Pasqua O. Use of prior knowledge and extrapolation in paediatric drug development: a case study with deferasirox. Eur J Pharm Sci. (2019) 136:104931. doi: 10.1016/j.ejps.2019.05.009, 31108206

[29] Oosterholt SP Della Pasqua O. Extrapolation and dosing recommendations for raxibacumab in children from birth to age <18 years. Br J Clin Pharmacol. (2021) 87:4709–17. doi: 10.1111/bcp.14893, 33974281

[30] Dimelow R Liefaard L Green Y Tomlinson R. Extrapolation of the efficacy and pharmacokinetics of Belimumab to support its use in children with lupus nephritis. Clin Pharmacokinet. (2024) 63:1313–26. doi: 10.1007/s40262-024-01422-y, 39320441 PMC11450137

[31] Healy P Allegaert K Della Pasqua O. Evaluation of the effect of CYP2D6 and OCT1 polymorphisms on the pharmacokinetics of tramadol: implications for clinical safety and dose rationale in paediatric chronic pain. Br J Clin Pharmacol. (2024) 91:283–96. doi: 10.1111/bcp.16201, 39384340 PMC11773095

[32] Page MJ McKenzie JE Bossuyt PM Boutron I Hoffmann TC Mulrow CD . The PRISMA 2020 statement: an updated guideline for reporting systematic reviews. J Clin Epidemiol. (2021) 134:178–89. doi: 10.1016/j.jclinepi.2021.03.001, 33789819

[33] Centers for Disease Control and Prevention (CDC). National Center for Health Statistics (NCHS). National Health and Nutrition Examination Survey Data. Hyattsville, MD: U.S. Department of Health and Human Services, Centers for Disease Control and Prevention. Available online at: https://wwwn.cdc.gov/nchs/nhanes/Default.aspx (Accessed April 12, 2024).

[34] Perkins BA Soleymanlou N Rosenstock J Skyler JS Laffel LM Liesenfeld KH . Low-dose empagliflozin as adjunct-to-insulin therapy in type 1 diabetes: a valid modelling and simulation analysis to confirm efficacy. Diabetes Obes Metab. (2020) 22:427–33. doi: 10.1111/dom.13945, 31858718 PMC7064984

[35] Mondick J Riggs M Sasaki T Sarashina A Broedl UC Retlich S. Mixed-effects modelling to quantify the effect of empagliflozin on renal glucose reabsorption in patients with type 2 diabetes. Diabetes Obes Metab. (2016) 18:241–8. doi: 10.1111/dom.12597, 26511213

[36] Mondick J Riggs M Kaspers S Soleymanlou N Marquard J Nock V. Population pharmacokinetic-pharmacodynamic analysis to characterize the effect of empagliflozin on renal glucose threshold in patients with type 1 diabetes mellitus. J Clin Pharmacol. (2018) 58:640–9. doi: 10.1002/jcph.1051, 29251772

[37] Baron KT Macha S Broedl UC Nock V Retlich S Riggs M. Population pharmacokinetics and exposure-response (efficacy and safety/tolerability) of empagliflozin in patients with type 2 diabetes. Diabetes Ther. (2016) 7:455–71. doi: 10.1007/s13300-016-0174-y, 27312794 PMC5014782

[38] Riggs MM Staab A Seman L MacGregor TR Bergsma TT Gastonguay MR . Population pharmacokinetics of empagliflozin, a sodium glucose cotransporter 2 inhibitor, in patients with type 2 diabetes. J Clin Pharmacol. (2013) 53:1028–38. doi: 10.1002/jcph.147, 23940010

[39] Riggs MM Seman LJ Staab A MacGregor TR Gillespie W Gastonguay MR . Exposure-response modelling for empagliflozin, a sodium glucose cotransporter 2 (SGLT2) inhibitor, in patients with type 2 diabetes. Br J Clin Pharmacol. (2014) 78:1407–18. doi: 10.1111/bcp.12453, 24964723 PMC4256629

[40] He N. Ethical considerations for clinical trials on medicinal products conducted with the paediatric population. Eur J Health Law. (2008) 15:223–50. doi: 10.1163/157180908x333228 18988606

[41] Howie SR. Blood sample volumes in child health research: review of safe limits. Bull World Health Organ. (2011) 89:46–53. doi: 10.2471/BLT.10.080010, 21346890 PMC3040020

[42] EFPIA MID3 Workgroup Marshall SF Burghaus R Cosson V Cheung SY Chenel M . Good practices in model-informed drug discovery and development: practice, application, and documentation. CPT Pharmacometrics Syst Pharmacol. (2016) 5:93–122. doi: 10.1002/psp4.12049, 27069774 PMC4809625

[43] Lyauk YK Jonker DM Lund TM. Dose finding in the clinical development of 60 US Food and Drug Administration-approved drugs compared with learning vs. confirming recommendations. Clin Transl Sci. (2019) 12:481–9. doi: 10.1111/cts.12641, 31254374 PMC6742935

[44] Beal SL Sheiner LB Boeckmann AJ Bauer RJ NONMEM 7.5 Users Guides 1989–2020 ICON plc Gaithersburg, MD, USA. Available online at: https://nonmem.iconplc.com/nonmem750/guides/ (Accessed April 12, 2024).

[45] Retout S Duffull S Mentré F. Development and implementation of the population fisher information matrix for the evaluation of population pharmacokinetic designs. Comput Methods Prog Biomed. (2001) 65:141–51. doi: 10.1016/S0169-2607(00)00117-6, 11275334

[46] Bauer RJ Hooker AC Mentre F. Tutorial for $DESIGN in NONMEM: clinical trial evaluation and optimization. CPT Pharmacometrics Syst Pharmacol. (2021) 10:1452–65. doi: 10.1002/psp4.12713, 34559958 PMC8674001

[47] R Core Team (2023). R Foundation for Statistical Computing, Vienna, Austria. Available online at: https://www.R-project.org/ (Accessed April 12, 2024).

[48] Li RJ Ma L Li F Li L Bi Y Yuan Y . Model-informed approach supporting drug development and regulatory evaluation for rare diseases. J Clin Pharmacol. (2022) 62:S27–37. doi: 10.1002/jcph.2143, 36461744

[49] Mitra A Tania N Ahmed MA Rayad N Krishna R Albusaysi S . New horizons of model informed drug development in rare diseases drug development. Clin Pharmacol Ther. (2024) 116:1398–411. doi: 10.1002/cpt.3366, 38989644

[50] de Castro FA Piana C Simões BP Lanchote VL Della Pasqua O. Busulfan dosing algorithm and sampling strategy in stem cell transplantation patients. Br J Clin Pharmacol. (2015) 80:618–29. doi: 10.1111/bcp.12648, 25819742 PMC4594698

[51] van Dijkman SC De Cock PAJG Smets K Decaluwe W Smits A Allegaert K 2019; 75:1393–1404, doi: DOI: 10.1007/s00228-019-02708-y, 31312867. 31312867

[52] Henschel AD Rothenberger LG Boos J. Randomized clinical trials in children–ethical and methodological issues. Curr Pharm Des. (2010) 16:2407–15. doi: 10.2174/138161210791959854, 20513232

[53] Sammons HM Starkey ES. Ethical issues of clinical trials in children. Paediatr Child Health. (2012) 22:47–50. doi: 10.1016/j.paed.2011.04.011

[54] Lava SAG Elie V Ha PTV Jacqz-Aigrain E. Sequential analysis in neonatal research-systematic review. Eur J Pediatr. (2018) 177:733–40. doi: 10.1007/s00431-018-3110-5, 29453599

[55] Bouazza N Dokoumetzidis A Knibbe CAJ de Wildt SN Ambery C De Cock PA . General clinical and methodological considerations on the extrapolation of pharmacokinetics and optimization of study protocols for small molecules and monoclonal antibodies in children. Br J Clin Pharmacol. (2022) 88:36256514:4985–96. doi: 10.1111/bcp.15571 36256514

[56] Garczarek U Muehlemann N Richard F Yajnik P Russek-Cohen E. Bayesian strategies in rare diseases. Ther Innov Regul Sci. (2023) 57:445–52. doi: 10.1007/s43441-022-00485-y, 36566312 PMC9789883

[57] Sailer MO Neubacher D Johnston C Rogers J Wiens M Pérez-Pitarch A . Pharmacometrics-enhanced Bayesian borrowing for pediatric extrapolation – a case study of the DINAMO trial. Ther Innov Regul Sci. (2024) 59:112–23. doi: 10.1007/s43441-024-00707-5, 39373938 PMC11706882

[58] Rascher J Cotton D Haertter S Brueckmann M. Clinical pharmacokinetics and pharmacodynamics of empagliflozin in patients with heart failure. Br J Clin Pharmacol. (2024) 90:2215–22. doi: 10.1111/bcp.16099, 38852615

[59] Laffel LMB Tamborlane WV Yver A Simons G Wu J Nock V . Pharmacokinetic and pharmacodynamic profile of the sodium-glucose co-transporter-2 inhibitor empagliflozin in young people with type 2 diabetes: a randomized trial. Diabet Med. (2018) 35:1096–104. doi: 10.1111/dme.13629, 29655290 PMC6099360

[60] Rascher J Cheng S Johnston C Härtter S Jan-Georg W Marquard J . Pharmacokinetics and pharmacodynamics of empagliflozin in paediatric patients aged 10-17 years with type 2 diabetes mellitus. Br J Clin Pharmacol. (2025) 91:2390–400. doi: 10.1002/bcp.70096, 40390307 PMC12304805

[61] Melin J Parkinson J Hamrén B Penland RC Boulton DW Tang W. Dapagliflozin pharmacokinetics is similar between patients with heart failure with reduced ejection fraction and patients with type 2 diabetes mellitus. Br J Clin Pharmacol. (2024) 90:606–12. doi: 10.1111/bcp.15939, 37897064

[62] Krekels EH Danhof M Tibboel D Knibbe CA. Ontogeny of hepatic glucuronidation; methods and results. Curr Drug Metab. (2012) 13:728–43. doi: 10.2174/138920012800840455, 22452455

[63] Alcorn J McNamara PJ. Ontogeny of hepatic and renal systemic clearance pathways in infants: part I. Clin Pharmacokinet. (2002) 41:959–98. doi: 10.2165/00003088-200241120-00003, 12222995

[64] Strassburg CP Strassburg A Kneip S Barut A Tukey RH Rodeck B . Developmental aspects of human hepatic drug glucuronidation in young children and adults. Gut. (2002) 50:259–65. doi: 10.1136/gut.50.2.259, 11788570 PMC1773113

