# Supplementary material for: Empagliflozin in paediatric heart failure: model-based optimisation of a pharmacokinetic bridging study
Source: Front Med (Lausanne). 2026 Feb 23;12:1522131. doi: 10.3389/fmed.2025.1522131 (PMC12968622; doi:10.3389/fmed.2025.1522131)
Supplement: Supplementary file 1 [file Data_Sheet_1.PDF]

frontiers

Supplementary Material

Supplementary table 1. Scenarios explored in the optimisation step.

Scenario	Title	# of participants	# of samples / participant	Detailed description
Optima 1.a	Hypothetical rich study	12	13	Visit 1: 12 samples<br>Visit 2: 1 sample
Optima 1.b	*Ibid.*	40	*Ibid.*	*Ibid.*
Optima 2	Traditional unoptimised	12	7	Visit 1: 6 samples<br>Visit 2: 1 sample
Optima 3	Optimised, 1 sampling scheme	12	7	Visit 1: 6 samples<br>Visit 2: 1 sample
Optima 4	Optimised, 2 sampling schemes	12	7 in 50%, 8 in 50% of participants	Visit 1: 6 samples<br>Visit 2: 1 sample<br>Visit 3: 1 sample in 50% of participants
Optima 5.a	Optimised, 4 sampling schemes	12	7 in 50%, 8 in 50% of participants	Visit 1: 6 samples<br>Visit 2: 1 sample<br>Visit 3: 1 sample in 50% of participants
Optima 5.b	*Ibid.*	*Ibid.*	7 to 10 (8 samples at Visit 1 if weight > 20kg, 50% having visit 3)	Visit 1: 8 samples if weight>20kg, otherwise 6 samples<br>Visit 2: 1 sample<br>Visit 3: 1 sample in 50% of participants
Optima 5.c	*Ibid.*	*Ibid.*	7	Visit 1: 6 samples<br>Visit 2: 1 sample
Optima 5.d	*Ibid.*	*Ibid.*	8	Visit 1: 6 samples<br>Visit 2: 1 sample<br>Visit 3: 1 sample (in all participants)
Optima 5.e	*Ibid.*	*Ibid.*	7	Visit 1: 5 samples<br>Visit 2: 1 sample<br>Visit 3: 1 sample (in all participants)
Optima 6	Optimised, 12 sampling schemes (individualized)	12	7 in 50%, 8 in 50% of participants	Visit 1: 6 samples<br>Visit 2: 1 sample<br>Visit 3: 1 sample in 50% of participants
Optima 7	Optimised, 3 samples at Visit 1	12	4	Visit 1: 3 samples<br>Visit 2: 1 sample
Optima 8	Optimised, 4 samples at Visit 1	12	5	Visit 1: 4 samples<br>Visit 2: 1 sample
Optima 9	Optimised, 5 samples at Visit 1	12	6	Visit 1: 5 samples<br>Visit 2: 1 sample
Optima 10	Optimised, 4 sampling schemes (= 5.a), but max 6h at Visit 1	12	7 in 50%, 8 in 50% of participants	Visit 1 (observation over max 6h): 6 samples<br>Visit 2: 1 sample<br>Visit 3: 1 sample in 50% of participants
Optima 11	Optimised, 4 sampling schemes, no Visit 3 (= 5.c), but max 6h at Visit 1	12	7	Visit 1 (observation over max 6h): 6 samples<br>Visit 2: 1 sample

Supplementary Table 2. Study characteristics and pharmacokinetic parameters of the identified population pharmacokinetic models on empagliflozin.

#	Reference	Disease	N	Dose range	Age	Weight	Bootstrap	VPC	CL (IIV CL) [L/h]	V_2 (IIV)	V_3 (IIV)	Q (IIV) [L/h]	K_a (IIV) [/h]	Lag-time	COV1	COV2	Comments
1	Perkins 2020	T1DM	1241	2.5-25mg OD	n.r.	n.r.	N	Y	n.r.	n.r.	n.r.	n.r.	n.r.	n.r.	n.r.	n.r.	
2	Mondick 2016	T2 DM	281	1, 2.5, 5, 10, 50, 100mg OD (single dose to 28 days)	57.8 ± 10.2	79.1 ± 19.5	Y	N	9.34 (0.0734)	4.70	72.4 (0.0637)	6.29 (0.0571)	1st order: 0.244 (0.0462) •Zero order D1: 0.544 (0.724)	0.158 (0.502)	BMI	BMI	2 compartment, sequential zero and first-order absorption with lag time. IIV: exp.
3	Mondick 2018	T1 DM	75	2.5 - 25mg OD	41 ± 10.9	79.3 ± 14.3	N	Y	12.3 (0.0387)	3.47	88.0 (0.077)	7.95 (0.042)	0.275 (0.028)	0.19	BMI	BMI	
4	Baron 2016	T2 DM	2761	1-100mg OD	58 ± 11.2		Y	N	10.6 (0.14)	3.14	70.6 (0.074)	6.34 (n.a.)	0.196 (0.026)	0.5	Age, eGFR, BMI, Sex, protein, Ex smoker, current smoker, ALT, Asian race	Age, Sex, Race, protein, BMI	
5	Riggs 2013	T2 DM	894	1, 2.5, 5, 10, 25, 50, 100mg OD	57.8 ± 12.5	85.1 ±23.8	Y	N	9.87 (26.9% = 0.0659)	3.02	60.4 (30.8% = 0.0686)	5.16 (n.a.)	0.224 (15.2% = 0.0545)	0.5 (FIX)	CL/F and Q/F: W	V2 and V3: W	
6	Riggs 2014	T2 DM	974	*Ibid.*	*Ibid.*	85 ±23	Y	N	*Ibid.*	*Ibid.*	*Ibid.*	*Ibid.*	*Ibid.*	*Ibid.*	Sex		

Abbreviations: T1 DM = type 1 diabetes mellitus, T2 DM = type 2 diabetes mellitus, VPC = visual predictive check(s), N = no, Y = yes, n.r. = not reported, CL = clearance, IIV = interindividual variability, V_2 = central volume of distribution, V_3 = peripheral volume of distribution, Q = intercompartmental clearance, K_a = absorption constant, COV1 = covariate on clearance(s), COV2 = covariate on volume(s) of distribution, BMI = body mass index, eGFR = estimated glomerular filtration rate, prot = total plasma protein concentration, ALT = alanine aminotransferase, W = body weight.

Supplementary Table 3: Results of the optimisation step through the $DESIGN function in Nonmem®. Blue = reference scenarios. Green = candidate scenarios. Red = excluded scenarios.

Scenario title	Optima 1.a	Optima 1.b	Optima 2	Optima 3	Optima 4	Optima 5.a	Optima 5.b	Optima 5.c	Optima 5.d	Optimisa 5.e	Optima 6	Optima 7	Optima 8	Optima 9	Optima 10	Opti[illegible]
Description	Optimisation 1 (rich: 12 samples at Visit 1, 1 sample at Visit 2)		Unoptimised	Optimised, 1 sampling scheme	Optimised, 2 sampling schemes	Optimised, 4 sampling schemes					Individualised sampling scheme	3 samples at Visit 1	4 samples at Visit 1	5 samples at Visit 1	Optimisation 5.a but Visit 1 max 6h	=Optimisation max 6h
Variants	12 patients	40 patients					8 samples at Visit 1 if W>20kg	No Visit 3	Visit 3 for all participants	5 samples at Visit 1, Visit 3 for all participants						
ΔOFV	-22.026	-31.663	-19.313	-20.248	-20.884	-20.299	-21.663	-19.885	-20.556	-20.857	-19.953	-16.556	-18.383	-19.508	-19.392	-18[illegible]
%RSE CL	8.11	4.44	8.35	8.21	8.20	8.21	8.17	8.22	8.18	8.17	8.20	8.49	8.32	8.23	8.24	8[illegible]
%RSE V2	21.3	17.6	33.4	31.7	31.1	30.9	29.8	31.7	31.1	31.9	32.6	39.1	34.9	33.2	33.0	3[illegible]
%RSE Q	17.3	9.46	20.0	18.3	17.8	19.2	17.2	19.2	18.8	17.8	19.4	21.6	20.3	19.6	20.8	2[illegible]
%RSE V3	11.6	6.22	12.9	12.0	11.5	11.9	11.3	12.2	12.1	11.1	12.5	14.5	12.9	12.0	14.4	1[illegible]
%RSE IIV CL	43.9	24.0	45.4	45.0	44.6	44.7	44.1	45.1	44.1	44.1	44.7	47.0	45.9	45.2	44.8	4[illegible]
%RSE IIV Q	243.4	135.2	318.7	282.0	268.0	303.0	246.9	306.5	287.2	273.2	301.2	376.5	341.5	322.2	303.0	30[illegible]
%RSE IIV V3	99.4	52.9	125.4	112.9	100.6	107.4	96.5	115.5	106.8	96.9	115.9	183.7	135.0	114.0	133.3	14[illegible]
%RSE Prop error	13.9	7.64	24.5	22.2	21.4	21.8	17.6	23.6	20.7	22.7	22.2	46.7	33.0	26.6	21.5	2[illegible]
EBV shrinkage CL (%)	6.28	3.16	9.04	8.43	7.66	7.86	7.01	8.68	6.93	6.92	7.87	11.8	10.1	8.89	8.33	9[illegible]
Shrinkage Q (%)	82.8	58.9	86.6	84.9	84.3	85.9	83.1	86.1	85.3	84.3	85.9	88.0	87.3	86.5	86.1	8[illegible]
Shrinkage V3 (%)	56.3	33.1	63.6	60.2	56.4	59.1	55.2	60.9	59.2	54.5	61.4	71.1	65.0	60.0	66.5	6[illegible]
Shrinkage k_a (%)	34.9	19.2	44.1	41.0	39.1	42.0	37.7	43.3	42.4	41.3	43.6	45.7	44.8	44.9	43.1	4[illegible]
Shrinkage D1 (%)	30.2	16.4	31.3	60.9	59.4	43.2	56.7	45.7	33.2	61.6	37.2	82.1	72.8	66.4	41.6	4[illegible]
Shrinkage ALAG1 (%)	71.6	47.0	71.7	95.0	94.8	86.7	94.4	85.6	82.1	95.1	75.6	97.7	96.6	95.7	86.1	8[illegible]

Supplementary Table 4: Sampling times for the individualized sampling scenario.

Participant identification number (ID)	ID1	ID2	ID3	ID4	ID5	ID6	ID7	ID8	ID9	ID10	ID11	ID12
Timing [h]												
Sample 1	0.44	0.99	0.68	0.52	0.42	0.70	0.69	0.46	0.42	0.44	1.03	0.43
Sample 2	1.07	2.17	0.92	0.94	0.98	0.92	0.92	1.00	1.06	1.23	1.03	1.02
Sample 3	2.38	2.27	2.25	2.19	2.25	2.00	2.15	2.19	1.88	2.10	2.21	2.57
Sample 4	4.00	3.97	4.03	3.97	4.09	2.72	4.04	4.07	3.96	3.99	4.05	4.03
Sample 5	7.18	7.03	6.87	6.67	7.01	4.06	6.87	6.50	7.96	7.11	6.71	6.99
Sample 6	7.93	7.92	7.96	7.94	7.89	7.93	7.96	7.96	168.05	7.96	7.84	7.96
Sample 7 (Visit 2)	168.09	168.15	167.87	167.96	168.07	167.73	167.91	168.08		168.07	167.85	168.05
Sample 8 (Visit 3)		501.18		503.13		503.47		503.21		501.13		502.54

Supplementary Table 5: Frequency of adverse events in adults (EMPEROR trial) and in children (systematic review), as well as expected number of trial participants (out of n=12 participants) experiencing them.

Adverse event	Adults with heart failure with reduced ejection fraction (HFrEF) from the EMPEROR trial [13], n (%)	Children (systematic review) [18], n (%)	Expected number of trial participants (out of n=12) experiencing the cited adverse event, n
Total participants exposed to empagliflozin	1863	268	12
Ketoacidosis or metabolic acidosis	0	0	0
Urinary tract infection	91 (4.9%)	11 (4.1%)	0 to 1
Genital infection	31 (1.7%)	4 (1.5%)	0 to 1
Hypoglycemia	27 (1.4%)	38 (14%)	0 to 2
Acute kidney injury	Not reported	4 (1.5%)	0 to 1

Supplementary Figures

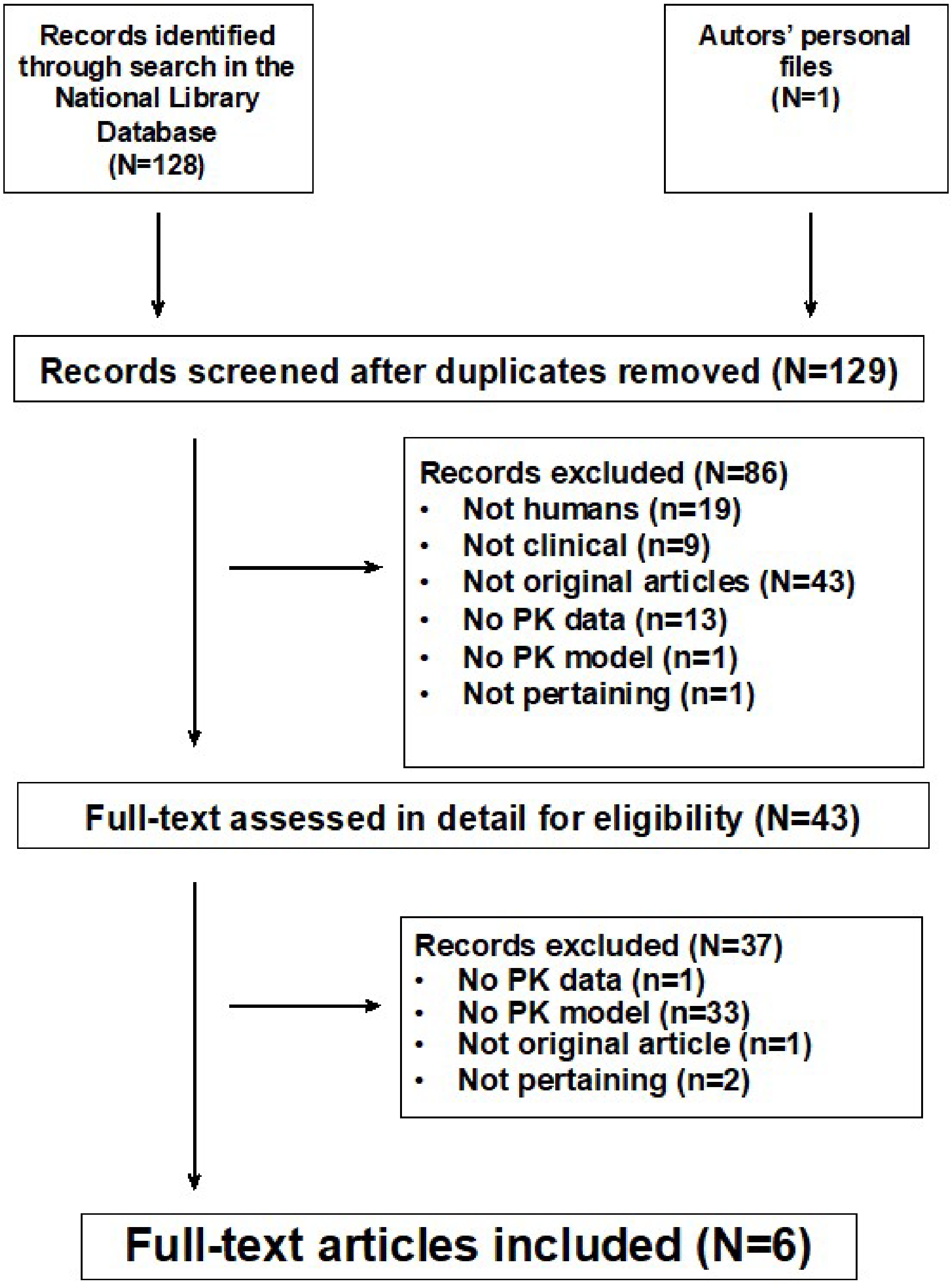

Supplementary Figure 1. Flowchart of the literature search process.

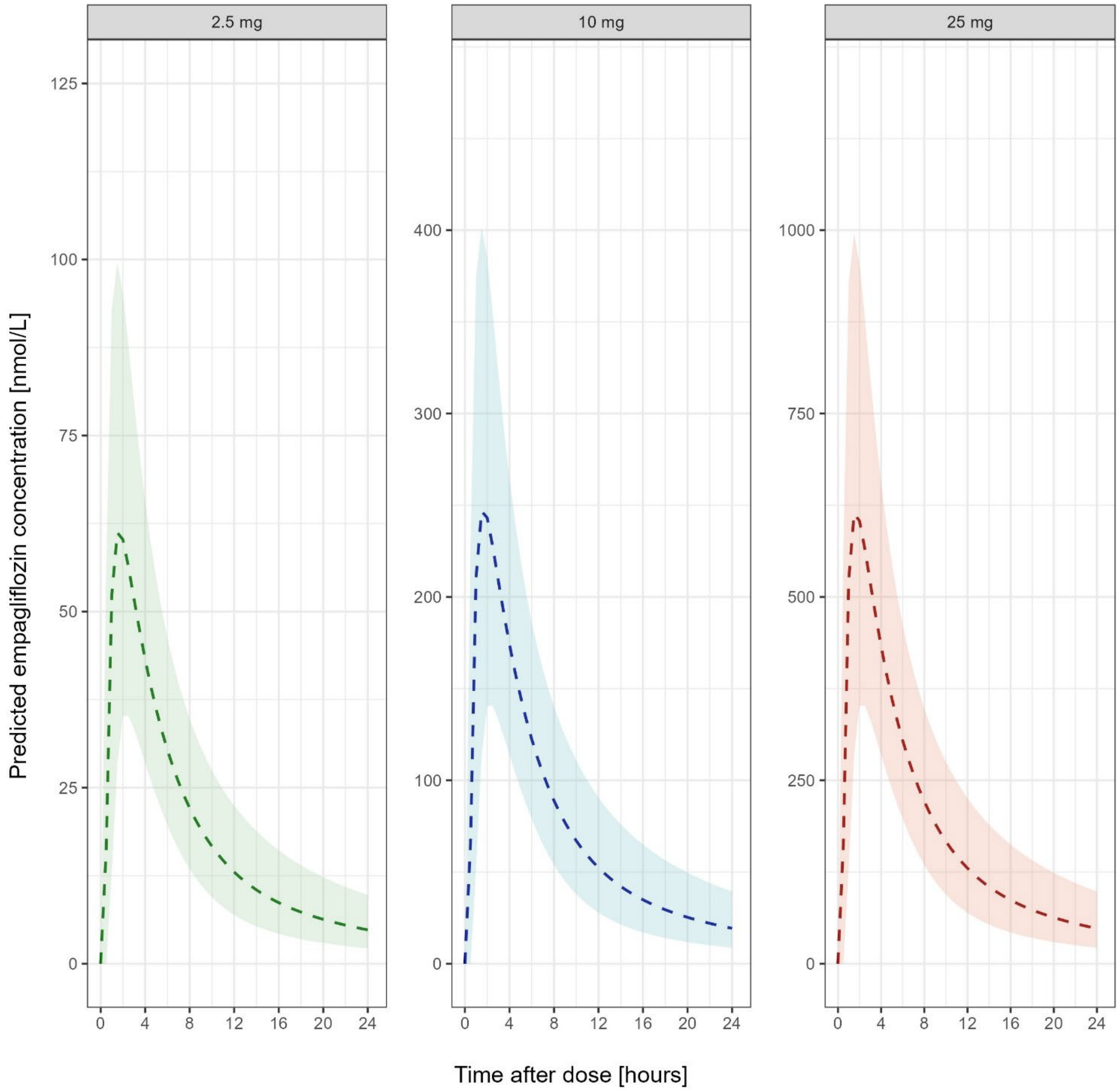

Supplementary Figure 2. Visual predictive checks of the original model, as run with an in-house reproduced NONMEM® script. The red lines represent the predicted mean concentration; the shaded areas depict the 95%-confidence interval of the predictions.

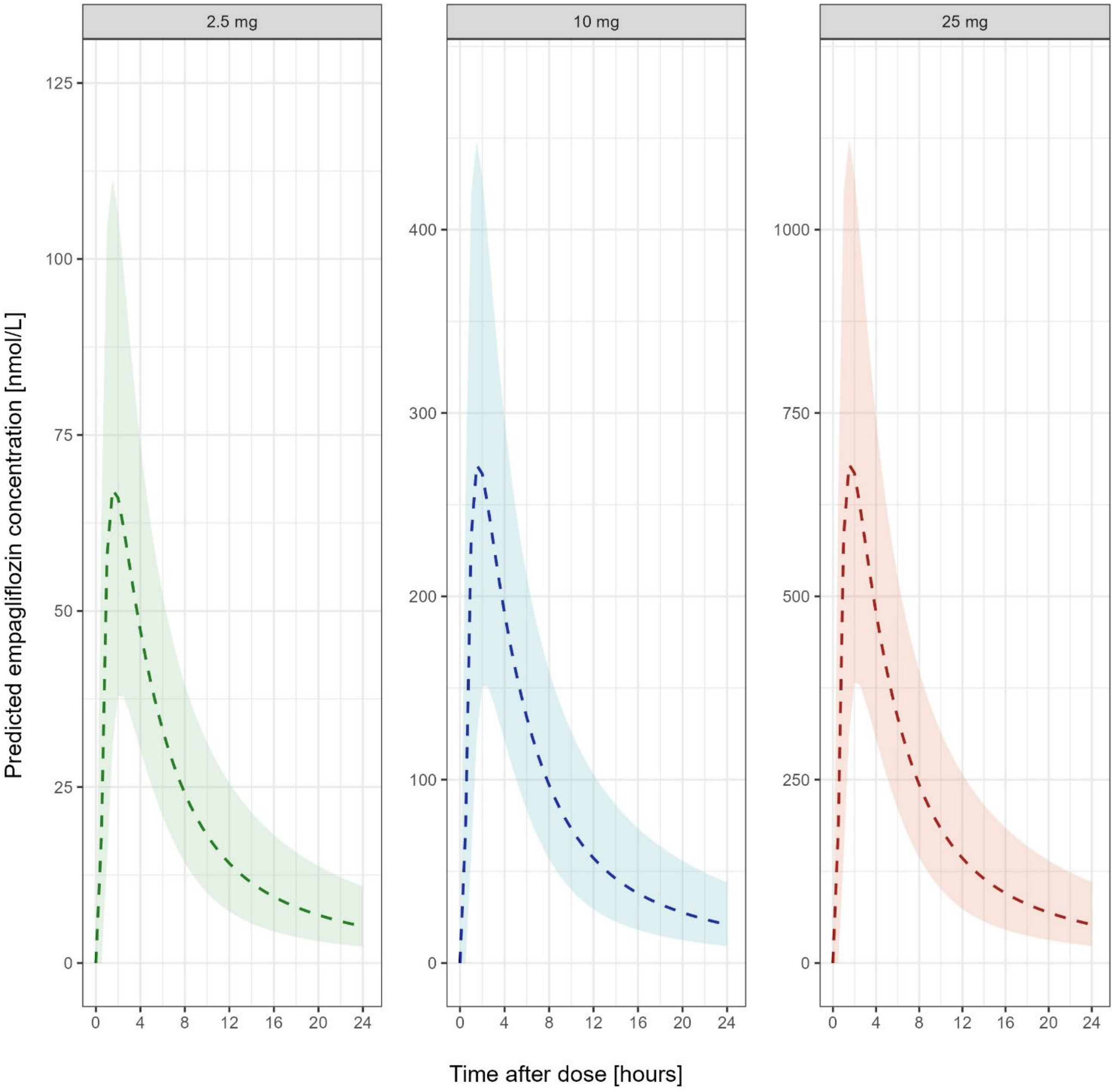

Supplementary Figure 3. Visual predictive checks of the reparametrized population pharmacokinetic model of empagliflozin, incorporating an allometric scaling, depicting the expected exposures of adult individuals getting 2.5mg, 10mg or 25mg once daily empagliflozin. The lines represent the predicted mean concentration, while the shaded areas depict the 95%-confidence interval of the predictions.

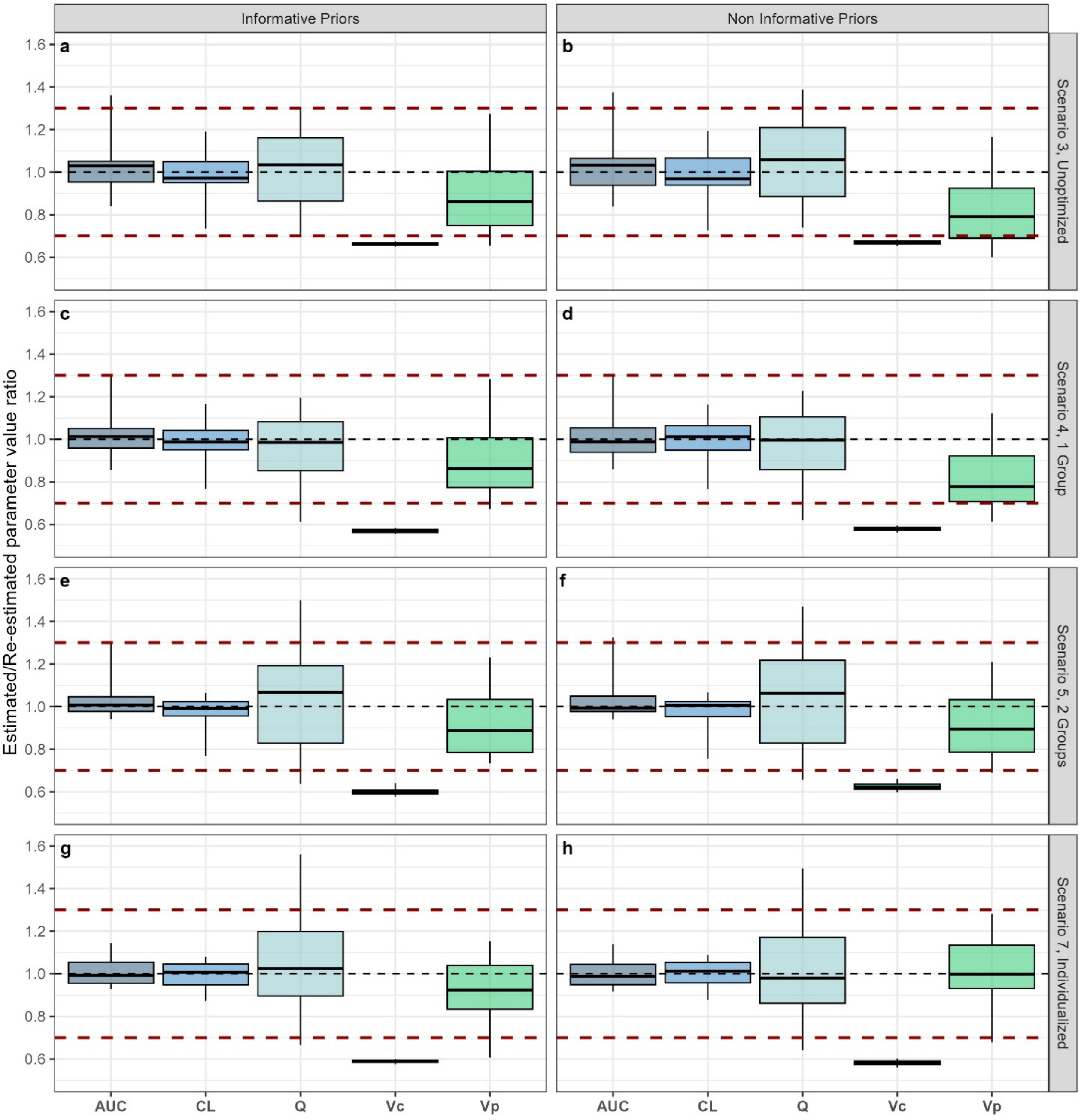

Supplementary Figure 4. Estimates of the primary and secondary pharmacokinetic parameters after simulation and re-estimation of 500 trials with 12 subjects each, according to the several simulated sampling schedules. The different sampling scenarios are detailed in Table 3. A well-performing sampling scheme would produce estimates close to 1.0 and with a relatively narrow distribution (in our case, we aimed for 0.7-1.3). Abbreviations: CL = clearance, V_c = central volume of distribution, V_p = peripheral volume of distribution, Q = intercompartmental clearance, AUC = area under the concentration-time curve.

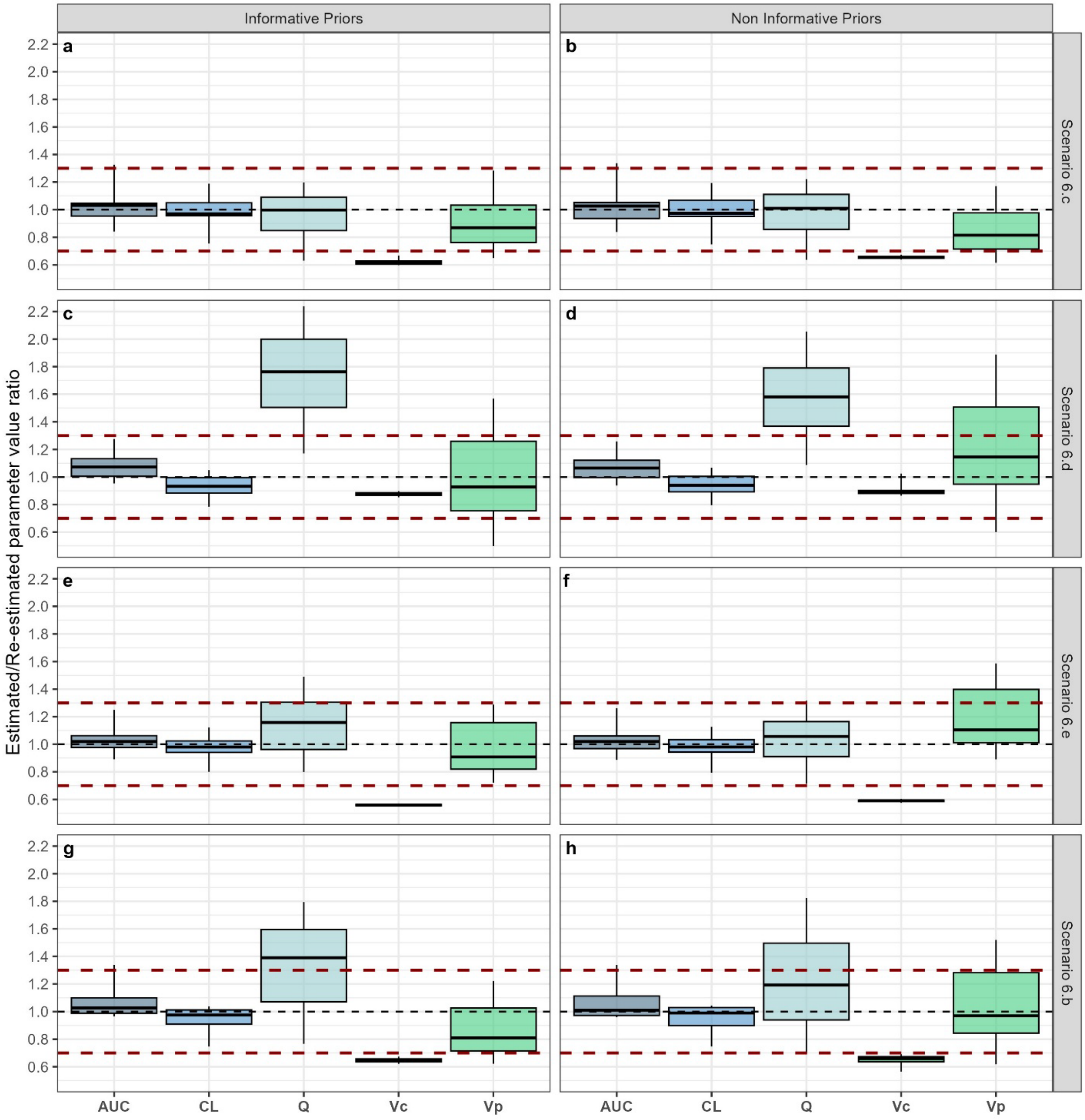

Supplementary Figure 5. Estimates of the primary and secondary pharmacokinetic parameters after simulation and re-estimation of 500 trials with 12 subjects each, according to the several simulated sampling schedules. The different sampling scenarios are explored variants of the chosen sampling scenario, depicted in Figure 5.e) and 5.f), and are detailed in Table 3. A well-performing sampling scheme would produce estimates close to 1.0 and with a relatively narrow distribution (in our case, we aimed for 0.7-1.3). Abbreviations: CL = clearance, V_c = central volume of distribution, V_p = peripheral volume of distribution, Q = intercompartmental clearance, AUC = area under the concentration-time curve.
